# Hypoxia-Induced Histone Lactylation Drives Cisplatin Resistance in Bladder Cancer by Promoting RBM15-Dependent m^6^A Methylation of IGFBP3

**DOI:** 10.34133/research.0970

**Published:** 2025-11-05

**Authors:** Jiazhu Sun, Yuchen Shi, Kai Yu, Suyuelin Huang, Dingheng Lu, Xinyang Niu, Yuxiao Li, Feifan Wang, Xueyou Ma, Jiangfeng Li, Xiaoyan Liu, Liping Xie, Haojie Huang, Ben Liu

**Affiliations:** ^1^ Department of Urology, The First Affiliated Hospital, Zhejiang University, School of Medicine, Hangzhou, 310003 Zhejiang, China.; ^2^ Institute of Urologic Science and Technology, The First Affiliated Hospital, Zhejiang University, School of Medicine, Hangzhou, 310003 Zhejiang, China.; ^3^ Department of Pathology, The First Affiliated Hospital, Zhejiang University, School of Medicine, Hangzhou, 310003 Zhejiang, China.; ^4^ Cancer Center, Zhejiang University, Hangzhou, 310003 Zhejiang, China.; ^5^ Ningbo Institute of Urology, Ningbo, 315042 Zhejiang, China.

## Abstract

Histone lactylation modification and RNA m^6^A modification play important roles in cisplatin resistance of bladder cancer (BCa). Hypoxia drives cisplatin resistance in BCa by analyzing the TCGA-BLCA cohort, where hypoxia signatures predicted poor overall survival. In vitro, hypoxia elevated lactate production via LDHA, inducing H3K18la catalyzed by KAT2B, which activated RBM15 transcription. RBM15 stabilized IGFBP3 mRNA via m^6^A modification depending on its SPOC domain, increasing IGFBP3 protein. Nuclear translocation of IGFBP3 complexed with p-EGFR/p-DNA-PKcs, enhancing DNA repair and reducing cisplatin-induced damage. Clinically, BCa tissues exhibited elevated LDHA/H3K18la/RBM15/IGFBP3, further amplifying post-cisplatin chemotherapy. Targeting this axis with LDHA inhibitor (stiripentol) and EGFR inhibitor (gefitinib) synergistically reversed cisplatin resistance in vitro and in vivo. This study unveils the “hypoxia–H3K18la–RBM15–IGFBP3” axis as a central driver of cisplatin resistance and proposes dual metabolic–epigenetic inhibition as a therapeutic strategy for refractory BCa.

## Introduction

Bladder cancer (BCa) remains a marked global health challenge, with cisplatin-based chemotherapy serving as a cornerstone for treatment [1]. However, the development of cisplatin resistance severely limits therapeutic efficacy, leading to poor patient outcomes [2]. Therefore, a substantial body of literature has conducted in-depth research on the treatment of cisplatin-resistant BCa and has achieved numerous marked results [3,4]. Tumor hypoxia, a hallmark of aggressive malignancies, is a well-documented driver of chemoresistance, yet the molecular mechanisms linking hypoxia to cisplatin resistance in BCa remain incompletely understood [5]. Previous studies showed that hypoxic conditions within solid tumors arise due to inadequate blood supply and rapid tumor cell proliferation, leading to reduced oxygen availability [6]. This hypoxic microenvironment activates adaptive cellular responses, primarily mediated by hypoxia-inducible factors (HIFs), which regulate genes involved in angiogenesis, metabolism, and survival [7]. Hypoxia prompts glycolytic flux and lactate accumulation via lactate dehydrogenase A (LDHA). Emerging evidence highlights lactate, a metabolic byproduct of hypoxia-induced glycolysis, as a key mediator of epigenetic reprogramming through histone lactylation—a novel posttranslational modification (PTM) implicated in gene regulation [8]. While lactylation has been associated with tumor progression, its role in modulating cisplatin resistance of BCa via hypoxia pathways remains unexplored.

Recent studies suggest that lactate not only fuels tumor metabolism but also regulates histone lactylation to alter transcriptional programs [9]. Lactylation, a newly discovered PTM, has emerged as a critical regulator of cellular processes in cancer. Lactylation involves the addition of lactate-derived lactyl groups to lysine residues on histone and nonhistone proteins, modulating their function and influencing gene expression [10]. Histone H3 lysine 18 lactylation (H3K18la) is the most common histone lactylation, which is usually enriched in the promoter region of genes and promotes gene transcription [8]. Recent studies have demonstrated that H3K18la can alter the expression of genes involved in drug efflux, DNA repair, and survival pathways, thereby promoting resistance to chemotherapeutic agents [11–16]. Understanding the role of lactylation in drug resistance provides new insights into the metabolic and epigenetic interplay in cancer and opens avenues for developing targeted therapies to overcome resistance. Previous studies have shown that H3K18la plays a role in promoting carcinogenesis in BCa and also contributes to cisplatin resistance [11,14]. However, whether hypoxia-driven lactylation contributes to cisplatin resistance in BCa by activating DNA repair pathways remains unknown.

The *N*^6^-methyladenosine (m^6^A) modification is the most abundant posttranscriptional modification on RNA, and it plays an important role in the occurrence and development of tumors as well as drug resistance [17]. The m^6^A “writer” complex composed of METTL3, METTL14, WTAP, and RBM15 is responsible for catalyzing the formation of m^6^A modification. Among them, METTL3 acts as a methyltransferase to synthesize the m^6^A modification, while RBM15 is responsible for binding to mRNA to enable its methylation [18]. RBM15 has been confirmed to promote the occurrence and development of various tumors, including acute myeloid leukemia, lung cancer, and prostate cancer [19–21]. There are also reports in BCa that it can promote the proliferation and migration of BCa [22,23]. However, no study has reported the relationship between RBM15 and histone lactylation. The role of RBM15 in cisplatin resistance of BCa has not been studied yet. Therefore, in this study, we explored the effect of hypoxia-regulated histone lactylation on the expression of RBM15 in cisplatin resistance of BCa.

The insulin-like growth factor-binding protein 3 (IGFBP3), a p53-regulated gene involved in DNA damage response, has been linked to chemoresistance in other cancers [24,25]. Previous studies have found that IGFBP3 can bind to epidermal growth factor receptor (EGFR) and DNA-dependent protein kinase catalytic subunit (DNA-PKcs) and promote their activation, thereby facilitating DNA damage repair [25–27]. However, its regulation by lactylation and role in BCa cisplatin resistance warrants investigation.

Here, we elucidate a hypoxia–lactylation–RBM15–IGFBP3 axis that drives cisplatin resistance in BCa. Through multi-omics analyses, functional assays, and clinical validation, we demonstrate that hypoxia-induced lactate accumulation enhances H3K18la via LDHA and the lactyltransferase KAT2B, promoting RBM15 expression. RBM15 promotes the formation of m^6^A on IGFBP3 mRNA, thereby enhancing its stability. IGFBP3 facilitates nuclear translocation of phosphorylated EGFR and DNA-PKcs, activating nonhomologous end-joining (NHEJ) repair to counteract cisplatin-induced DNA damage. This study unveils a previously unrecognized mechanism of hypoxia-mediated cisplatin resistance and provides actionable targets to overcome therapeutic failure in BCa.

## Results

### Hypoxia induces cisplatin resistance in BCa

**Fig. 1.**
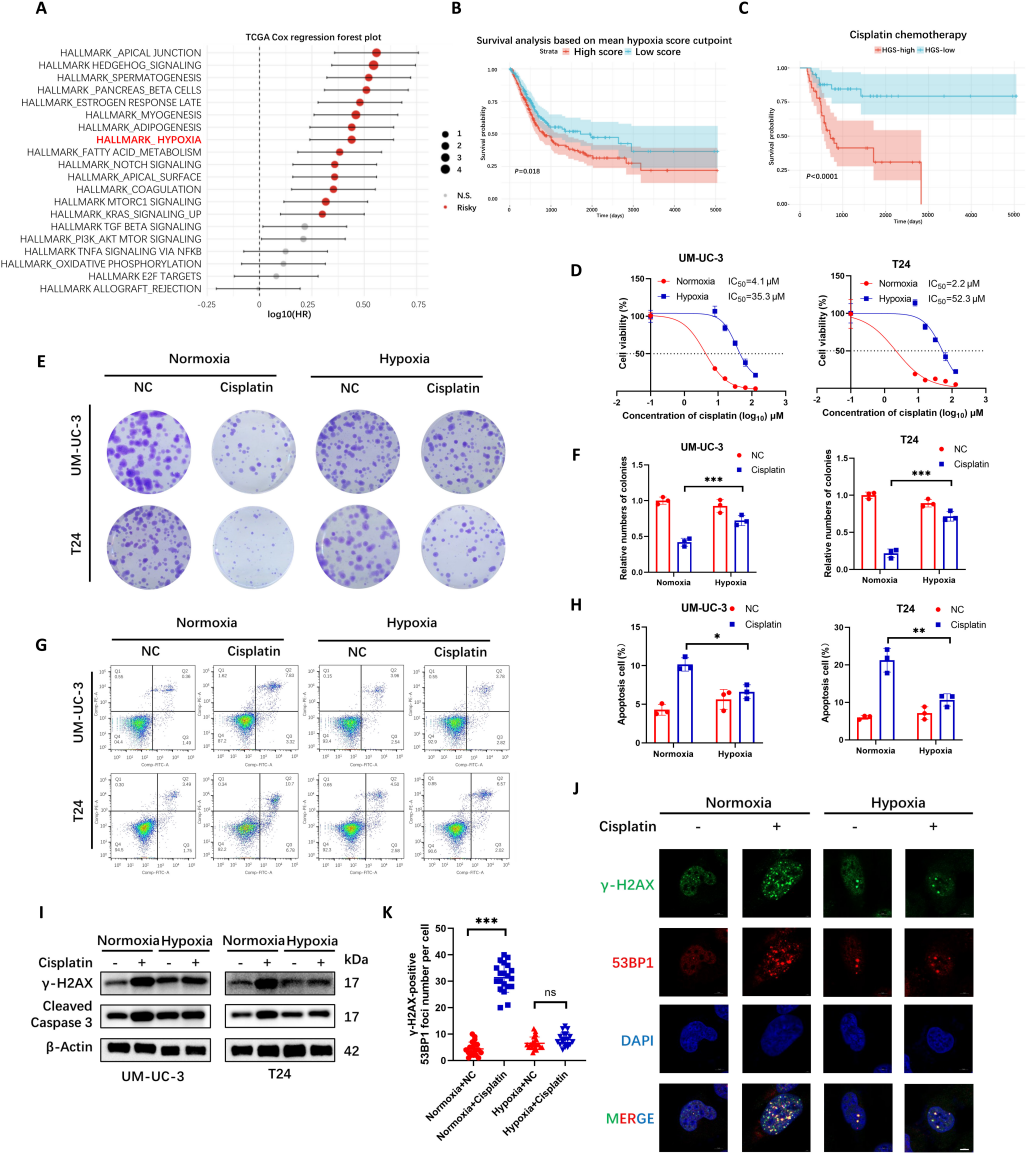
Hypoxia induces cisplatin resistance in BCa. (A) Forest plot of Cox regression analysis for hypoxia-related pathways in TCGA-BCa patients. (B) Kaplan–Meier OS curves comparing high- versus low-hypoxia score groups. (C) Kaplan–Meier OS curves for HGS-high and HGS-low BCa patients post-cisplatin treatment. (D) IC_50_ values of cisplatin in UM-UC-3 and T24 cells cultured under normoxia (20% O_2_) or hypoxia (1% O_2_). (E) Colony formation assays evaluating cisplatin (2 μM) sensitivity under normoxia (20% O_2_) or hypoxia (1% O_2_). (F) Quantification of numbers of colony. (G) Flow cytometry analysis of apoptosis in cisplatin-treated (2 μM) cells under normoxia (20% O_2_) or hypoxia (1% O_2_). (H) Quantification of apoptosis cells. (I) Western blot analysis of γ-H2AX and cleaved caspase 3 levels in cisplatin-treated (2 μM) cells under normoxia (20% O_2_) or hypoxia (1% O_2_). (J) IF of γ-H2AX and 53BP1 in cisplatin-treated (2 μM) cells under normoxia (20% O_2_) or hypoxia (1% O_2_). (K) Quantification of 53BP1 foci number in γ-H2AX-positive cells. Scale bar, 5 μm. *P < 0.05; **P < 0.01; ***P < 0.001; ns, not significant.

In order to explore the relationship between the expression of hypoxia-related genes and the prognosis of BCa patients, we analyzed the RNA-sequencing (RNA-seq) data of BCa patients in The Cancer Genome Atlas (TCGA) database. RNA-seq data from 401 BCa samples in the TCGA cohort were analyzed using gene set variation analysis (GSVA). Univariate Cox regression revealed a significant association between poor overall survival (OS) and hypoxia-related pathways (Fig. 1A). Patients were stratified into high- and low-hypoxia score groups, with Kaplan–Meier analysis confirming significantly worse OS in the high-hypoxia group (Fig. 1B), establishing hypoxia as a key prognostic risk factor in BCa. Weighted gene coexpression network analysis (WGCNA) of whole-transcriptome profiles identified that coexpression modules correlated with hypoxia scores (Fig. S1A). The red and black modules, showing the strongest hypoxia association (Fig. S1B and C), were selected for further study. Least absolute shrinkage and selection operator (LASSO) Cox regression analysis (λ = 0.064) refined these modules to 16 prognostic genes, which were used to construct a hypoxia-related gene signature (HGS) (Fig. S1D and E). Individual HGS scores were calculated as the weighted sum of these genes’ expression levels (Fig. S1F). Using the median HGS score as a cutoff, patients were classified into HGS-high or HGS-low subgroups. Gene Ontology (GO) and Kyoto Encyclopedia of Genes and Genomes (KEGG) analysis was enriched in hypoxia-related pathways (Fig. S1G and H). The HGS-high group exhibited significantly shorter OS (Fig. S1I). Clinically, high HGS scores correlated with advanced tumor/normal (T/N) stages and higher pathological grades (Fig. S1J). Strikingly, HGS-high patients showed markedly worse OS following cisplatin chemotherapy (Fig. 1C), suggesting hypoxia-mediated cisplatin resistance. To verify the relationship between hypoxia and cisplatin resistance in BCa, UM-UC-3 and T24 cells were treated in a hypoxic environment (1% O_2_) for 24 h to detect their IC_50_ (median inhibitory concentration) values under cisplatin treatment. The results showed that BCa cells after 24 h of hypoxia are more resistant than in the normoxic environment (Fig. 1D). The plate colony formation experiment indicated that BCa cells in the hypoxic environment had a stronger resistance to cisplatin-induced cell proliferation inhibition than those in the normoxic environment (Fig. 1E and F). The flow cytometry apoptosis experiment showed that BCa cells in the hypoxic environment had a stronger resistance to cisplatin-induced apoptosis than those in the normoxic environment (Fig. 1G and H). Besides, BCa cells in the hypoxic environment had a stronger ability to resist cisplatin-induced γ-H2AX and cleaved caspase 3 elevation than those in the normoxic environment, suggesting that hypoxia can reduce cisplatin-induced DNA damage and apoptosis in BCa (Fig. 1I). 53BP1 is recruited to DNA damage sites by γ-H2AX to facilitate DNA repair and serves as a critical biomarker for assessing the extent of DNA damage. Immunofluorescence (IF) analysis revealed that the number of cisplatin-induced 53BP1 and γ-H2AX colocalized foci is significantly lower under hypoxic conditions compared to normoxic conditions (Fig. 1J and K). The above results indicate that the expression of hypoxia-related genes is related to the prognosis of the patient and hypoxia causes cisplatin resistance in BCa.

### Hypoxia-derived lactate promotes cisplatin resistance via H3K18la up-regulation

**Fig. 2.**
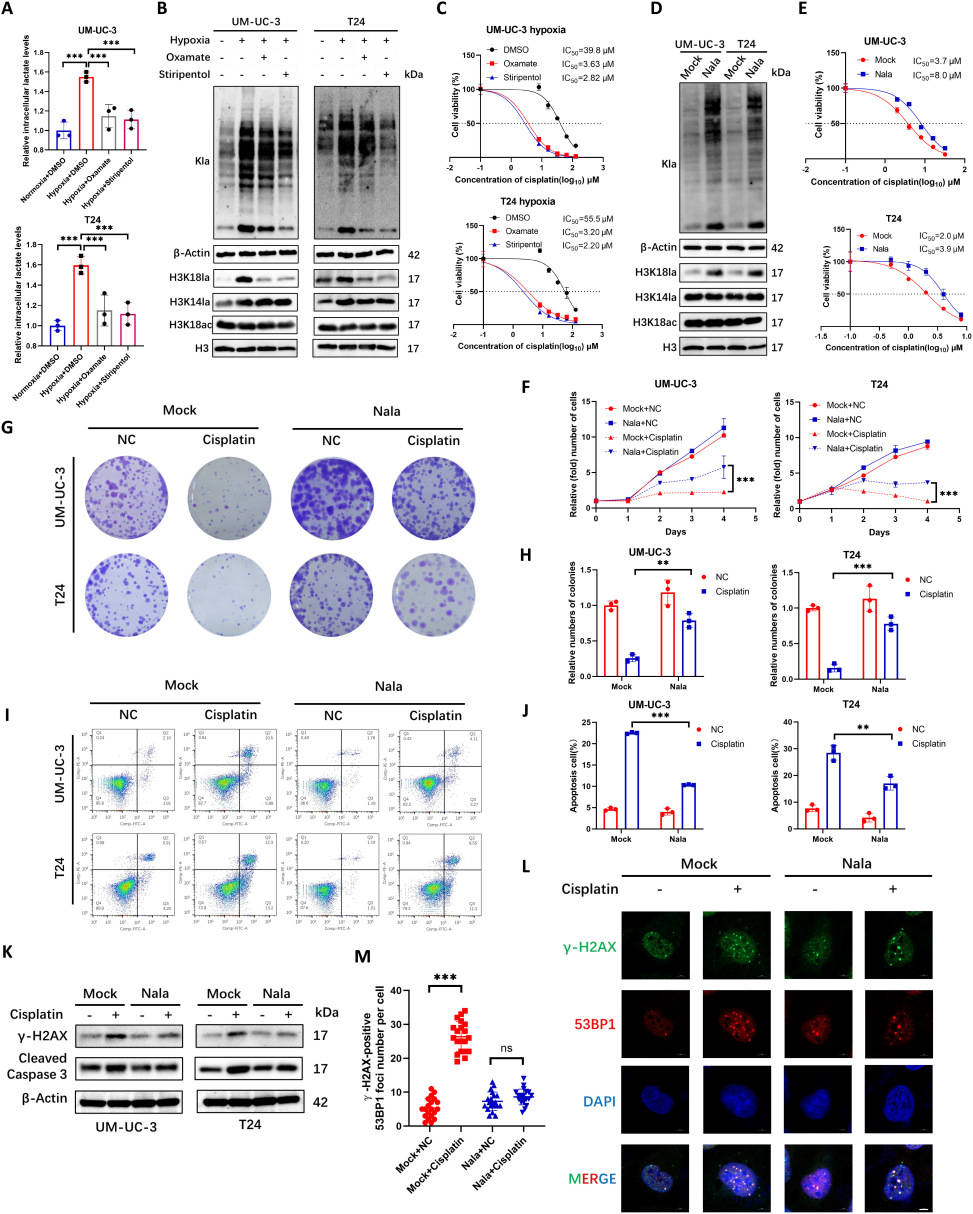
Hypoxia-derived lactate promotes cisplatin resistance via H3K18la up-regulation. (A to C) l-Lactate levels (A), Western blot analysis of lactylation levels (B), and IC_50_ values (C) in UM-UC-3 and T24 cells under normoxia (20% O_2_) or hypoxia (1% O_2_), with LDHAi oxamate (20 mM) and stiripentol (100 μM). (D and E) Western blot analysis of lactylation levels (D) and IC_50_ values (E) of Nala-treated (20 mM) cells. (F) CCK-8 assay of cisplatin sensitivity with/without Nala (20 mM). (G) Colony formation assays of cisplatin-treated (2 μM) cells with/without Nala (20 mM). (H) Quantification of numbers of colony. (I) Apoptosis analysis of cisplatin-treated (2 μM) cells with/without Nala (20 mM). (J) Quantification of apoptosis cells. (K) γ-H2AX and cleaved caspase 3 levels in cisplatin-treated (2 μM) cells with/without Nala (20 mM). (L) IF of γ-H2AX and 53BP1 in cisplatin-treated (2 μM) cells with/without Nala (20 mM). (M) Quantification of 53BP1 foci number in γ-H2AX-positive cells. Scale bar, 5 μm. **P < 0.01; ***P < 0.001; ns, not significant.

Hypoxia can lead to an increase in glycolysis in cells, thereby causing lactate accumulation. Therefore, to explore the mechanism by which hypoxia induces cisplatin resistance in BCa cells, we measured the lactate content and lactylation levels in BCa cells under 0-, 12-, 24-, and 48-h hypoxic conditions, confirming lactate accumulation peaks and lactylation peaks at 24 h (Fig. S2A and B). LDHA, as a key enzyme in cellular glycolysis, plays an important role in catalyzing pyruvate to lactate (Fig. S2C). Under the action of LDHA inhibitors (LDHAi) oxamate and stiripentol, the cell viability decreased (Fig. S2D). Furthermore, our assays demonstrated that LDHA inhibition treatment under hypoxic conditions significantly attenuates the intracellular lactate accumulation and the elevation of global protein lactylation induced by hypoxia. Moreover, hypoxia-induced lactylation primarily targets the histone mark H3K18la, with no significant effects observed on H3K14la or H3K18ac levels (Fig. 2A and B). Meanwhile, using oxamate and stiripentol in a hypoxic environment could reduce cisplatin resistance of BCa (Fig. 2C). Then, we added 0, 10, 20, and 40 mM sodium lactate(Nala) to BCa cells to increase the lactate levels in BCa cells and found that 20 mM Nala treatment induces a peak intracellular lactylation level in cells (Fig. S2E). Furthermore, Nala treatment predominantly enhances the level of H3K18la while exhibiting minimal impact on H3K14la and H3K18ac (Fig. 2D). Additionally, Nala treatment also confers enhanced cisplatin resistance in BCa cells (Fig. 2E). Moreover, the lactate levels, global lactylation levels, and histone lactylation H3K18la levels in BCa cells increased after cisplatin treatment (Fig. S2F and G). This indicates that lactylation levels are related to cisplatin resistance in BCa. The plate colony formation assay and Cell Counting Kit-8 (CCK-8) assay showed that Nala enhanced the cisplatin resistance of BCa cells against cell proliferation inhibition caused by cisplatin (Fig. 2F to H). Flow cytometry apoptosis experiments showed that Nala enhanced the cisplatin resistance of BCa cells against cell apoptosis caused by cisplatin (Fig. 2I and J). Also, Nala enhanced the ability of BCa cells to resist γ-H2AX and cleaved caspase 3 elevation caused by cisplatin, indicating that the increase in intracellular lactylation levels can reduce cisplatin-induced DNA damage and apoptosis in BCa (Fig. 2K). IF analysis also revealed that the number of cisplatin-induced 53BP1 and γ-H2AX colocalized foci is significantly lower with Nala treatment compared to negative control (Fig. 2L and M). These results indicate that cisplatin resistance of BCa caused by hypoxia is caused by promoting the increase in lactylation levels in BCa cells.

### KAT2B enhances the expression of RBM15 by facilitating the formation of H3K18la under hypoxic conditions

**Fig. 3.**
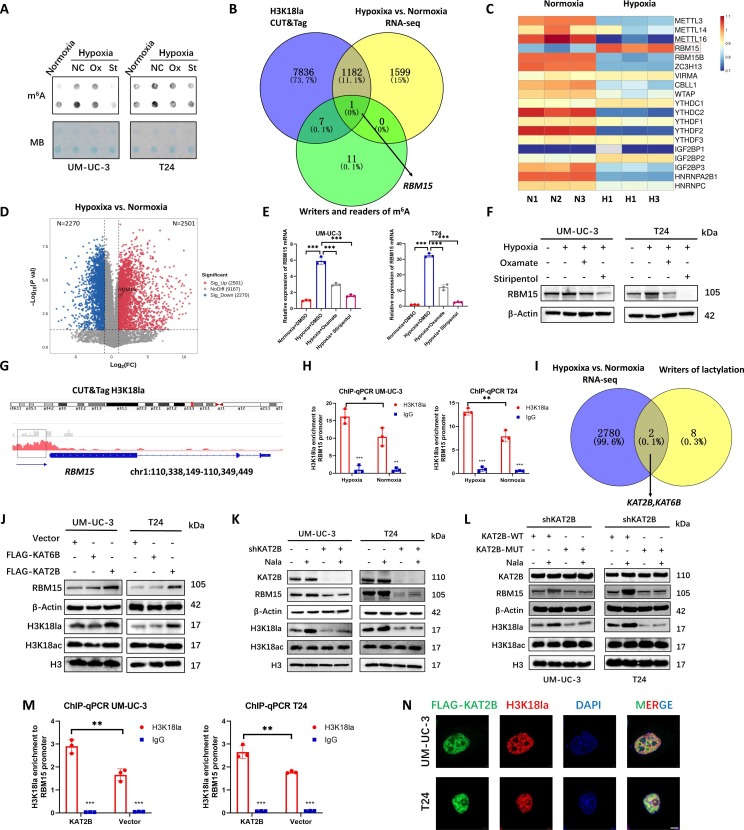
KAT2B enhances the expression of RBM15 by facilitating the formation of H3K18la under hypoxic conditions. (A) Dot blot analysis showing RNA m^6^A levels under normoxia (20% O_2_) or hypoxia (1% O_2_), with LDHAi oxamate (20 mM) and stiripentol (100 μM). (B) Venn diagram of overlapping genes up-regulated by hypoxia (1% O_2_), enriched for H3K18la at promoters (CUT&Tag) and writers and readers of m^6^A modification. (C) Heatmap showing expression of writers and readers of m^6^A modification in normoxia (20% O_2_)- or hypoxia (1% O_2_)-exposed UM-UC-3 cells. (D) Volcano plots of DEGs in hypoxia-exposed UM-UC-3 cells. (E and F) RT-qPCR analysis of RBM15 mRNA (E) and Western blot analysis of RBM15 protein levels (F) under normoxia (20% O_2_) or hypoxia (1% O_2_), with LDHAi oxamate (20 mM) and stiripentol (100 μM). (G) H3K18la peaks at the RBM15 promoter. (H) ChIP-qPCR of H3K18la binding to the RBM15 promoter under normoxia/hypoxia. (I) Overlap between hypoxia-up-regulated genes and lactylation-associated writers. (J) Western blot analysis of KAT6B and KAT2B overexpression. (K) Western blot analysis of KAT2B and Nala (20 mM) effects on H3K18la, H3K18ac, and RBM15. (L) Western blot analysis of wild-type KAT2B (KAT2B-WT) and mutated KAT2B (KAT2B-MUT) with Nala (20 mM). (M) ChIP-qPCR of H3K18la binding after KAT2B overexpression. (N) IF staining of FLAG-KAT2B (green), H3K18la (red), and nuclei (DAPI, blue) under hypoxia (1% O_2_). Scale bar, 5 μm. *P < 0.05; **P < 0.01; ***P < 0.001.

Previous studies have found that hypoxia and lactylation are both associated with m^6^A modification, and they play an important role in tumor drug resistance [12,28,29]. Through dot blot experiments, we found that the level of RNA m^6^A modification increased in BCa under hypoxic conditions, and the m^6^A level decreased after adding the LDHAi (Fig. 3A). To investigate the specific mechanism by which hypoxia regulates downstream gene expression through histone lactylation H3K18la in BCa, we employed RNA-seq to detect alterations in gene expression within BCa cells following hypoxia treatment. Gene expression under hypoxia condition with *P* < 0.05 and |log_2_FC| > 1 was screened. The results showed that the expression of 2,501 genes was significantly up-regulated after hypoxia treatment (Fig. 3B and D). In our previous research, through cleavage under targets and tagmentation (CUT&Tag) technology, we detected that the promoter regions of 9,025 genes were enriched with H3K18la in BCa (Fig. 3B) [14]. By taking the intersection of these 2 datasets, we identified 1,183 genes whose promoter regions were enriched with H3K18la, and H3K18la promoted the expression of these genes (Fig. 3B). KEGG pathway analysis revealed that these genes were enriched in pathways such as the HIF-1 signaling pathway and transcriptional misregulation in cancer pathway (Fig. S3A). Among these genes, only RBM15 is a gene related to m^6^A modification (Fig. 3B). The heatmap RNA-seq shows the expression change levels of various m^6^A-related genes after hypoxia in BCa cells (Fig. 3C). In the TCGA database, the expression levels of LDHA in BCa patients were positively correlated with that of RBM15 (Fig. S3B). Our detection indicated that both the mRNA and protein expression levels of RBM15 increased under hypoxic condition and decreased after treatment with the LDHAi oxamate and stiripentol (Fig. 3E and F). Using the IGV software to visualize CUT&Tag data, we found that, compared with immunoglobulin G (IgG), H3K18la was highly enriched in the promoter region of the RBM15 gene (Fig. 3G). Chromatin immunoprecipitation (ChIP)–quantitative polymerase chain reaction (qPCR) results demonstrated that H3K18la was enriched in the promoter region of RBM15, and the degree of enrichment increased after hypoxia (Fig. 3H). To explore the lactyltransferase that contributes to the hypoxia-mediated elevation of H3K18la, we screened genes that were highly expressed in RNA-seq following hypoxia. Among the known lactyltransferases (P300, CBP, KAT2A, KAT2B, KAT5, KAT6B, KAT8, AARS1 AARS2, and ATAT1), KAT2B and KAT6B were found to be highly expressed (Fig. 3I). After overexpressing KAT2B and KAT6B separately in BCa cells, we found that KAT2B could promote the expression of H3K18la and RBM15, while KAT6B did not exhibit this function (Fig. 3J). H3K18ac was not regulated by KAT2B or KAT6B (Fig. 3J). In the TCGA database, the expression level of KAT2B in BCa patients was positively correlated with that of RBM15 (Fig. S3C). Furthermore, adding Nala to BCa cells increased the expression levels of H3K18la and RBM15. However, when KAT2B was knocked down, adding Nala failed to enhance the levels of H3K18la and RBM15, indicating that the formation of H3K18la relies on KAT2B to catalyze Nala for lactylation modification (Fig. 3K). H3K18ac was not regulated by Nala and KAT2B (Fig. 3K). We further constructed a KAT2B plasmid (KAT2B-MUT) in which the Y616 and F617 sites were mutated to A616 and A617, respectively. This mutation led to the loss of lactylation catalytic activity of the expressed KAT2B protein (Fig. S3D). We overexpressed the wild-type and mutant forms of KAT2B in BCa cells with KAT2B knockdown. The results showed that the mutant KAT2B could not catalyze the formation of H3K18la, unlike the wild-type KAT2B (Fig. 3L). H3K18ac was not regulated by mutant or wild-type KAT2B (Fig. 3L). The ChIP-qPCR experiment also verified that KAT2B could promote the enrichment of H3K18la in the promoter region of RBM15 (Fig. 3M). The IF experiment revealed that KAT2B and H3K18la were colocalized within the nuclei of BCa cells (Fig. 3N).

### RBM15 promotes cisplatin resistance in BCa by stabilizing IGFBP3 mRNA via m^6^A modification

**Fig. 4.**
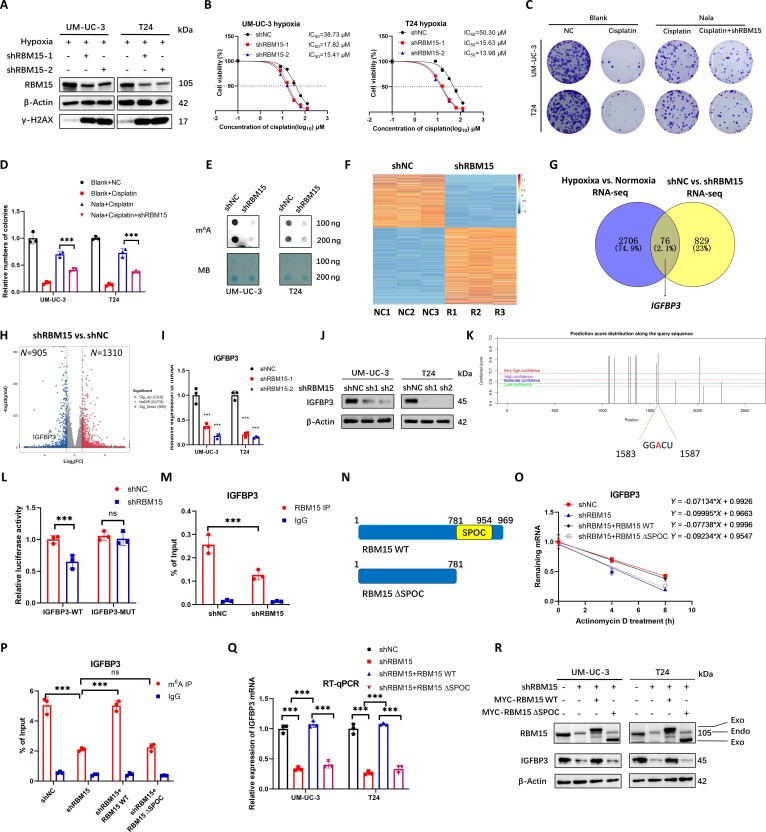
RBM15 promotes cisplatin resistance in BCa by stabilizing IGFBP3 mRNA via m^6^A modification. (A) Western blot analysis of γ-H2AX levels in RBM15-knockdown (shRBM15) cells under hypoxia (1% O_2_). (B) IC_50_ values of shNC and shRBM15 cells under hypoxia (1% O_2_). (C) Colony formation assays of cisplatin (2 μM) or Nala (20 mM) cells with or without RBM15 knockdown. (D) Quantification of numbers of colony. (E) Dot blot of RNA m^6^A levels in cells with or without RBM15 knockdown. (F) Heatmaps showing gene expression of UM-UC-3 with or without RBM15 knockdown. (G) Venn diagram of overlapping genes up-regulated by hypoxia and down-regulated by RBM15 knockdown. (H) Volcano plots of DEGs in RBM15-knockdown UM-UC-3 cells. (I and J) RT-qPCR analysis of IGFBP3 mRNA (I) and Western blot analysis of IGFBP3 protein levels (J) in RBM15-knockdown cells. (K) Predicted sites in IGFBP3 mRNA in the SRAMP database. (L) Relative luciferase activities of IGFBP3-WT of IGFBP3-MUT in UM-UC-3 cells with RBM15 knockdown. (M) RIP-qPCR assays showing the binding of RBM15 with IGFBP3 mRNA with or without RBM15 knockdown. (N) Schematic diagram of RBM15 WT and RBM15 ΔSPOC protein. (O) RBM15 knockdown reduced IGFBP3 mRNA half-life in UM-UC-3 cells. (P to R) MeRIP-qPCR analysis of m^6^A enrichment (P), RT-qPCR analysis of IGFBP3 mRNA (Q), and Western blot analysis of IGFBP3 protein levels (R) in RBM15-knockdown cells overexpressing RBM15WT and RBM15 ΔSPOC. ***P < 0.001; ns, not significant.

To investigate the function of RBM15 in BCa, we quantified the expression levels of RBM15 in BCa cell lines and observed a significant up-regulation compared to the SV-HUC-1 human normal uroepithelial cell line, with particularly pronounced elevation in UM-UC-3 and T24 cell lines (Fig. S4A). The IC_50_ value decreased with RBM15 knockdown in UM-UC-3 and T24 cells under hypoxic conditions, and the expression level of γ-H2AX increased (Fig. 4A and B). This indicates that RBM15 knockdown under hypoxic conditions will cause an increase in DNA damage in BCa and an increase in the sensitivity of BCa to cisplatin. The plate colony formation experiment showed that sodium lactate can reduce the resistance of BCa to cisplatin by RBM15 knockdown (Fig. 4C and D). To further explore the role of KAT2B and RBM15 in cisplatin resistance of BCa, we found that overexpression of RBM15 in BCa after cisplatin treatment could restore the increase in γ-H2AX expression caused by KAT2B knockdown (Fig. S4B). Overexpression of RBM15 in BCa cells could also restore the weakened colony formation ability and decreased IC_50_ value caused by KAT2B knockdown (Fig. S4C to E). As an m^6^A methyltransferase, RBM15 knockdown can decrease the m^6^A level of RNA in BCa cells (Fig. 4E). To explore the downstream genes regulated by RBM15, we performed RNA-seq of BCa cells in the negative control (shNC) group and shRBM15 group (Fig. 4F). We found that the expression levels of 905 genes decreased after RBM15 knockdown (Fig. 4G and H). Taking the intersection of these genes with the genes up-regulated after hypoxia, there were 76 genes that were simultaneously regulated by hypoxia and RBM15 (Fig. 4G). Among these genes, IGFBP3 caught our attention. In the TCGA database, the expression level of IGFBP3 in BCa patients was positively correlated with RBM15 (Fig. S4F). After RBM15 knockdown, the mRNA and protein levels of IGFBP3 in BCa cells decreased significantly (Fig. 4I and J). Furthermore, under hypoxic conditions, the expression of IGFBP3 increases. Sodium lactate can rescue the decrease in IGFBP3 caused by RBM15 knockdown under hypoxic conditions (Fig. S4G). Further, we used the online prediction website for m^6^A sites (http://www.cuilab.cn/sramp) to predict the m^6^A sites on IGFBP3 mRNA (Fig. 4J). We constructed the sequences of the wild-type (IGFBP3-WT) and mutant (IGFBP3-MUT) m^6^A sites of IGFBP3 in a reporter plasmid containing the luciferase gene (Fig. S4H). The luciferase dual-reporter assay proved that the fluorescence intensity of BCa cells transfected with IGFBP3-WT plasmid after RBM15 knockdown was significantly lower than that of the shNC group, while there was no significant change in the fluorescence intensity between the shNC group and shRBM15 group transfected with IGFBP3-MUT plasmid (Fig. 4L). The RNA immunoprecipitation (RIP) experiment proved that RBM15 directly binds to the mRNA of IGFBP3, and the binding level decreased after RBM15 knockdown (Fig. 4M). The SPOC domain in RBM15 is crucial for formation of m^6^A modification. We constructed wild-type RBM15 plasmid (RBM15 WT) and truncated SPOC domain RBM15 plasmid (RBM15 ΔSPOC) (Fig. 4N). The RNA stability experiment proved that the stability of IGFBP3 mRNA decreased after RBM15 knockdown, and the stability could be rescued by RBM15 WT rather than RBM15 ΔSPOC (Fig. 4O). The methylated RNA immunoprecipitationqPCR (MeRIP-qPCR) experiment showed that the m^6^A level on IGFBP3 decreased after RBM15 knockdown, while the m^6^A level increased after overexpression of RBM15 WT, and there was no significant change after overexpression of RBM15 ΔSPOC (Fig. 4P). This indicates that RBM15 impacts the formation of m^6^A modification on IGFBP3 mRNA through the SPOC domain. Simultaneously, after RBM15 knockdown, the mRNA and protein levels of IGFBP3 decreased; after overexpressing RBM15 WT, the mRNA and protein levels increased; and after overexpressing RBM15 ΔSPOC, there were no significant changes in the mRNA and protein levels (Fig. 4Q and R). Consistent with these findings, colony formation assays further demonstrated that overexpression of RBM15 WT enhances cisplatin resistance in BCa cells, whereas the RBM15 ΔSPOC fails to confer such resistance (Fig. S4I and J). The above results indicate that RBM15 promotes cisplatin resistance in BCa, and RBM15 impacts the formation of m^6^A modifications on the IGFBP3 mRNA through the SPOC domain to maintain its stability.

### IGFBP3 drives cisplatin resistance in BCa

**Fig. 5.**
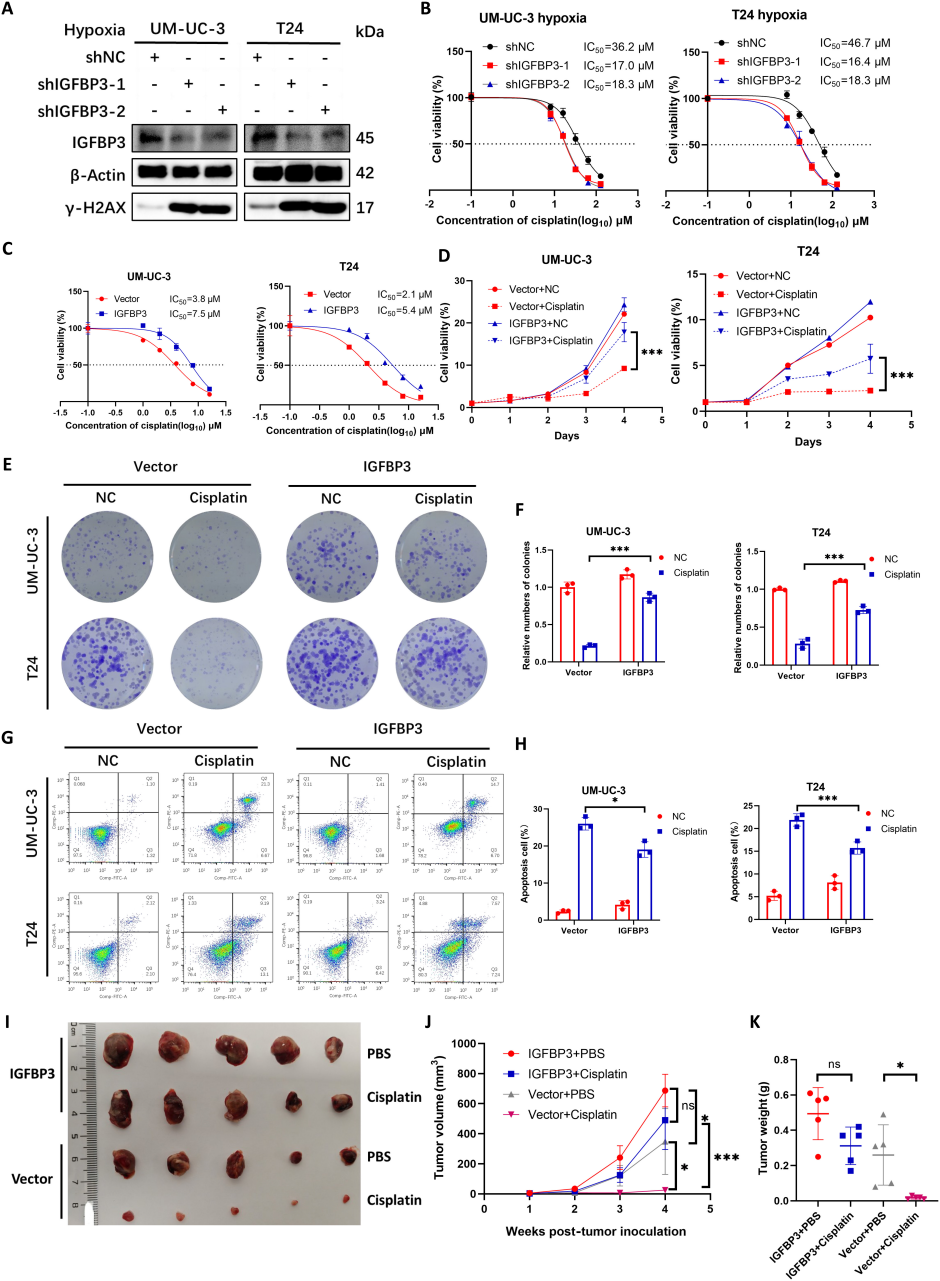
IGFBP3 drives cisplatin resistance in BCa. (A) Western blot analysis of γ-H2AX levels in IGFBP3-knockdown (shIGFBP3) cells under hypoxia (1% O_2_). (B) IC_50_ values of shNC and shIGFBP3 cells under hypoxia (1% O_2_). (C) IC_50_ values of control and IGFBP3-overexpressing cells. (D) CCK-8 assay of cisplatin sensitivity with or without IGFBP3 overexpression. (E) Colony formation assays of cisplatin-treated (2 μM) cells with or without IGFBP3 overexpression. (F) Quantification of numbers of colony. (G) Apoptosis analysis of cisplatin-treated (2 μM) cells with or without IGFBP3 overexpression. (H) Quantification of apoptosis cells. (I to K) Subcutaneous xenograft model: tumor size (I), growth curves (J), and weights (K) in mice inoculated with overexpressing vector or IGFBP3 cells treated with cisplatin (5 mg/kg every 3 d) or PBS. *P < 0.05; ***P < 0.001; ns, not significant.

In the TCGA database, the expression level of IGFBP3 in BCa tissues was significantly higher than that in normal bladder tissues (Fig. S5A and B), and it was also highly expressed in patients with lymph node metastasis (Fig. S5C). At the same time, the OS period of BCa patients with high expression of IGFBP3 was significantly shorter than that of patients with low expression (Fig. S5D). In order to explore the function of IGFBP3 in BCa, BCa cells under hypoxic conditions were found, where their IC_50_ value decreased following IGFBP3 knockdown, while the expression level of γ-H2AX increased (Fig. 5A and B). The use of LDHAi can also reduce the high expression of IGFBP3 caused by hypoxia in BCa (Fig. S5E). This indicates that IGFBP3 knockdown under hypoxic conditions will increase the DNA damage degree of BCa and increase the sensitivity of BCa to cisplatin. Overexpression of IGFBP3 in BCa increases its IC_50_ value (Fig. 5C). CCK-8 assay and plate colony formation assay showed that overexpression of IGFBP3 enhanced the resistance of BCa cells to cisplatin-induced cell proliferation inhibition (Fig. 5D to F). Flow cytometry apoptosis assay indicated that overexpression of IGFBP3 enhanced the resistance of BCa cells to cisplatin-induced apoptosis (Fig. 5G and H). Subcutaneous tumor formation experiments in nude mice also showed that IGFBP3 can promote cisplatin resistance in BCa in vivo (Fig. 5I to K). In summary, we have verified in vivo and in vitro that IGFBP3 can increase the cisplatin resistance of BCa.

### Hypoxia promotes IGFBP3 nuclear transport and activates EGFR and DNA-PKcs

**Fig. 6.**
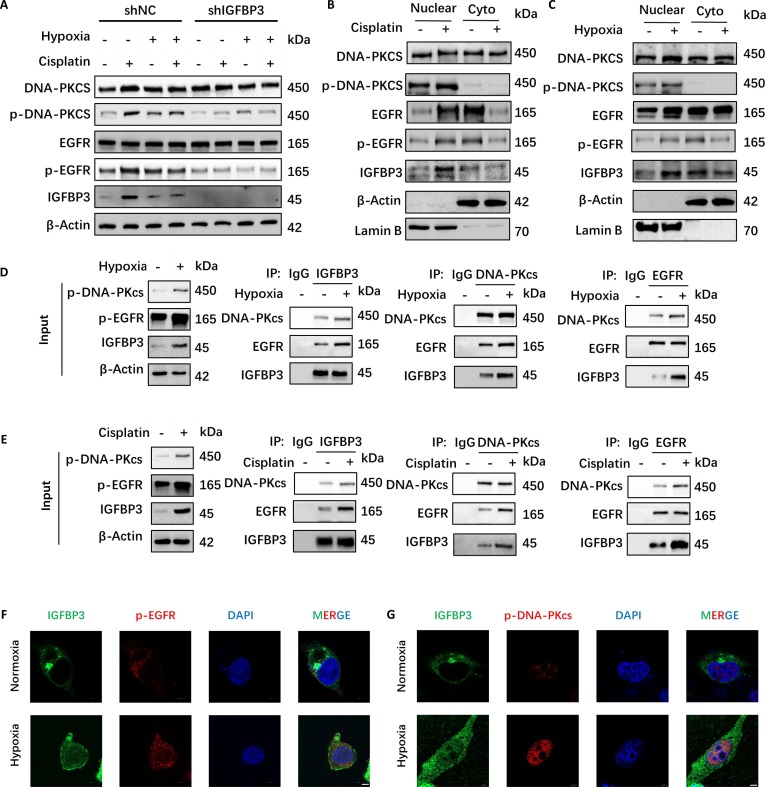
Hypoxia promotes IGFBP3 nuclear transport and activates EGFR and DNA-PKcs. (A) Western blot analysis of IGFBP3, p-EGFR (Tyr^1068^), and p-DNA-PKcs (Thr^2609^) in shNC and shIGFBP3 cells under cisplatin (2 μM)/hypoxia (1% O_2_). (B and C) Western blot analysis of subcellular localization of IGFBP3, p-EGFR, and p-DNA-PKcs in cisplatin-treated (2 μM) (B) or hypoxic (1% O_2_) (C) cells. (D and E) Co-IP of EGFR, DNA-PKcs, and IGFBP3 in cisplatin-treated (2 μM) (D) or hypoxic (1% O_2_) (E) cells. (F) IF staining of IGFBP3 (green) and p-EGFR (red). (G) IF staining of IGFBP3 (green) and p-DNA-PKcs (red). Scale bar, 5 μm.

Previous studies have reported that IGFBP3 can promote the phosphorylation of EGFR and DNA-PKcs by binding to them, and facilitate their nuclear translocation. Phosphorylated DNA-PKcs promotes NHEJ, thereby causing the resistance of paclitaxel to breast cancer [25]. We found that the expression of IGFBP3 in BCa cells increased after they were treated with cisplatin, and the expressions of phosphorylated EGFR and DNA-PKcs also increased (Fig. 6A). Under hypoxic conditions, the expression levels of IGFBP3, p-EGFR, and p-DNA-PKcs caused by cisplatin were lower than those under normoxic conditions (Fig. 6A). This indicates that the degree of DNA damage caused by cisplatin in hypoxic environments is lower than that in normoxic environments. The expression levels of p-EGFR and p-DNA-PKcs decreased in both hypoxic or cisplatin-treated conditions compared to negative control following IGFBP3 knockdown (Fig. 6A). This suggests that the phosphorylation of EGFR and DNA-PKcs depends on IGFBP3. In addition, by extracting proteins from the nuclei and cytoplasm of BCa cells for Western blot experiments, we found that IGFBP3, EGFR, and DNA-PKcs all translocated to the nucleus after cisplatin treatment or in hypoxic environments, and the proportion of p-EGFR and p-DNA-PKcs in the nucleus also increased (Fig. 6B and C). Co-immunoprecipitation (IP) experiments also showed that the binding ability between IGFBP3, EGFR, and DNA-PKcs was enhanced in hypoxic environments or under the action of cisplatin (Fig. 5D and E). IF experiments also indicated that hypoxia caused IGFBP3, p-EGFR, and p-DNA-PKcs to translocate to the nucleus, and IGFBP3, p-EGFR, and p-DNA-PKcs were colocalized in the nucleus (Fig. 5F and G). Cisplatin also had the same effect (Fig. S6A and B). These results indicate that hypoxia promotes the binding of IGFBP3 and EGFR, DNA-PKcs, and their phosphorylation and nuclear translocation, thereby resisting DNA damage caused by cisplatin.

### The combination of LDHAi and EGFRi can promote cisplatin sensitivity of cisplatin-resistant BCa cells

**Fig. 7.**
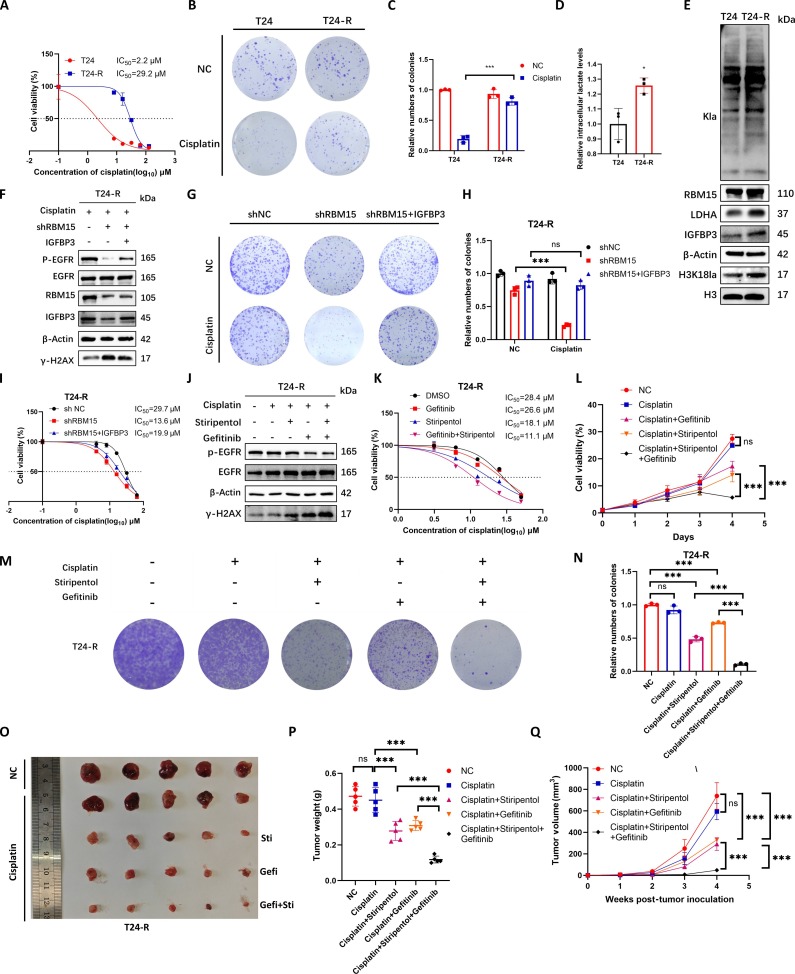
The combination of LDHAi and EGFRi can promote cisplatin sensitivity of cisplatin-resistant BCa cells. (A) IC_50_ values of cisplatin in T24-R (resistant) versus T24 (parental) cells. (B) Colony formation assays of cisplatin-treated (2 μM) T24 and T24-R cells. (C) Quantification of numbers of colony. (D) l-Lactate levels in T24 versus T24-R cells. (E) Western blot analysis of Kla, RBM15, H3K18la, LDHA, and IGFBP3 levels in T24-R versus T24 cells. (F) Western blot analysis of RBM15-knockdown T24-R cells overexpressing IGFBP3. (G) Colony formation assays in RBM15-knockdown T24-R cells overexpressing IGFBP3. (H) Quantification of numbers of colony. (I) IC_50_ values of RBM15-knockdown T24-R cells overexpressing IGFBP3. (J) Western blot analysis of T24-R cells treated with cisplatin (2 μM), gefitinib (1 μM), and stiripentol (100 μM). (K) IC_50_ values of T24-R cells treated with gefitinib (1 μM) and stiripentol (100 μM). (L) CCK-8 assay of T24-R cells treated with cisplatin (2 μM), gefitinib (1 μM), and stiripentol (100 μM). (M) Colony formation assays of T24-R cells treated with cisplatin (2 μM), gefitinib (1 μM), and stiripentol (100 μM). (N) Quantification of numbers of colony. (O to Q) Xenograft tumor size (O), growth curves (P), and weights (Q) in mice inoculated with T24-R cells treated with cisplatin (3 mg/kg), stiripentol (150 mg/kg), gefitinib (10 mg/kg), or PBS twice weekly. Data are mean ± SD (*n* = 3). *P < 0.05; ***P < 0.001; ns, not significant.

In order to explore a new therapeutic strategy for BCa with cisplatin resistance by targeting the H3K18la/IGFBP3/EGFR/DNA-PKcs signaling axis, we constructed a cisplatin-resistant BCa cell line T24-R by cisplatin induction. Through CCK-8 assay, it was found that the IC_50_ value of T24-R was higher than that of the T24 cell line (Fig. 7A), and the plate colony formation assay verified that the cisplatin resistance of the T24-R cell line was stronger than that of the T24 cell line (Fig. 7B and C). The lactate level detection showed that the lactate content in the T24-R cells was higher than that in the T24 cells (Fig. 7D). Western blot assay revealed that the Kla, H3K18la, LDHA, RBM15, and IGFBP3 levels in the T24-R cells were higher than those in the T24 cells (Fig. 7E). Under cisplatin treatment, the overexpression of IGFBP3 in T24-R cells effectively reverses the elevation of γ-H2AX levels induced by RBM15 knockdown (Fig. 7F). Furthermore, overexpression of IGFBP3 can rescue the weakened platelet colony-forming ability and the decreased IC_50_ observed in T24-R cells after RBM15 knockdown (Fig. 7G to I). To explore drugs that reverse cisplatin resistance in BCa, we used EGFR inhibitor (EGFRi) gefitinib and LDHAi stiripentol in BCa cells to enhance the effect of cisplatin. We found that the combined use of gefitinib and stiripentol was more effective in promoting the increase of γ-H2AX levels in T24-R cells caused by cisplatin compared to using either drug alone (Fig. 7J). The combination of gefitinib and stiripentol could more significantly reduce the IC_50_ value compared to using either drug alone (Fig. 7K). The CCK-8 assay and plate colony formation assay also indicated that the inhibitory effect of cisplatin on the proliferation ability of T24-R cells was not significant, while the proliferation ability of cells decreased after adding gefitinib or stiripentol, and the combination of gefitinib and stiripentol could further reduce the proliferation ability (Fig. 7L to N). To further explore the effect of gefitinib and stiripentol combination in reversing cisplatin resistance in vivo, we conducted subcutaneous tumor formation experiments in nude mice using T24-R cells. Body weight measurements performed at weekly intervals revealed no significant differences among the various groups of nude mice, indicating that the administered drug dosages were within a safe and tolerable range (Fig. S7A). It was found that the inhibitory effect of cisplatin on subcutaneous tumors in nude mice was not significant, while the tumor volume and weight decreased after intraperitoneal injection of gefitinib and stiripentol, and the combination of gefitinib and stiripentol could further reduce the tumor volume and weight (Fig. 7O to Q). Immunohistochemical (IHC) staining of subcutaneous tumors in nude mice revealed that the combined administration of gefitinib and stiripentol significantly reduced Ki67 expression within the tumor tissues (Fig. S7B and C). In summary, IGFBP3 knockdown in T24-R cisplatin-resistant BCa cells can reverse their resistance, and the combination of EGFRi and LDHAi can reverse their cisplatin resistance.

### Clinical validation of histone lactylation, RBM15I, and IGFBP3 in BCa

**Fig. 8.**
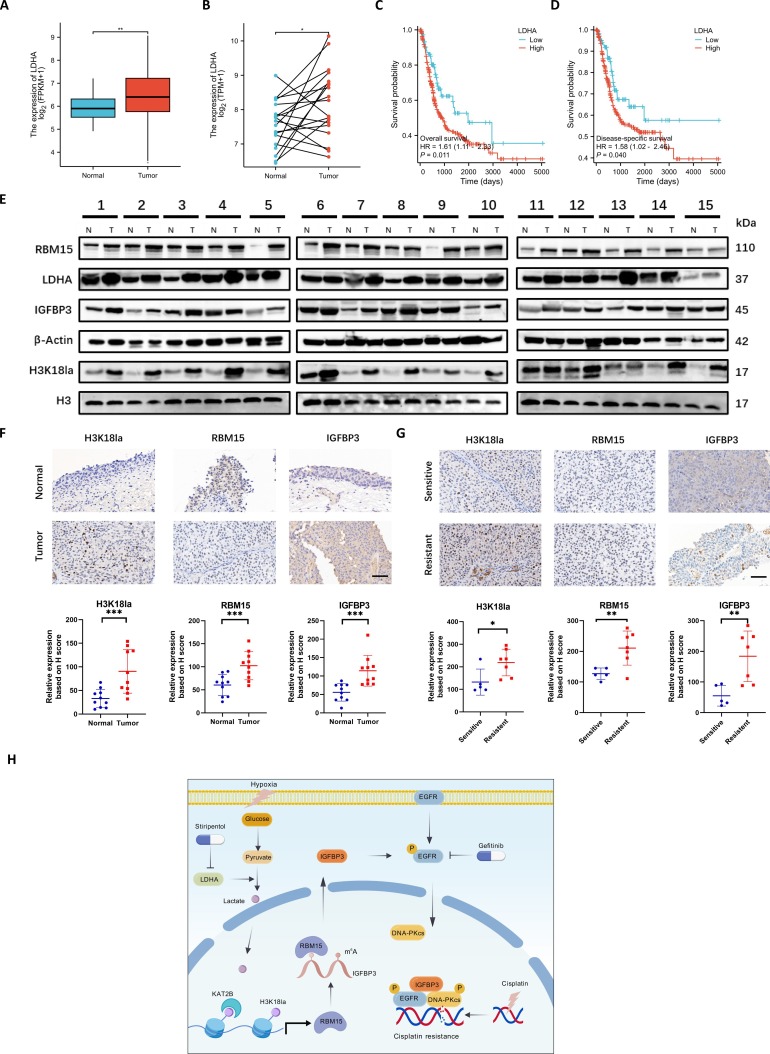
Clinical validation of histone lactylation, RBM15, and IGFBP3 in BCa. (A and B) LDHA expression in unpaired (A) and paired (B) BCa tissues in TCGA database. (C and D) Kaplan–Meier survival curves for patients with high versus low LDHA. (E) Western blot analysis of H3K18la, RBM15, LDHA, and IGFBP3 in 15 matched BCa (T) and normal (N) tissues. (F and G) IHC staining of H3K18la, LDHA, and RBM15 in tumor/adjacent tissues (F) and cisplatin-sensitive/resistant samples (G). Scale bar, 60 μm. (H) Schematic summary of hypoxia-driven cisplatin resistance. *P < 0.05; **P < 0.01; ***P < 0.001.

In order to further explore the expression patterns of histone lactylation-related proteins in clinical samples of BCa patients, we first analyzed the expression patterns of LDHA in BCa from the TCGA database. The results showed that LDHA was highly expressed in tumor tissues in both nonmatched and matched BCa patient tissue samples (Fig. 8A and B). Moreover, the OS rate and disease-specific survival rate were lower in the patients with high expression of LDHA (Fig. 8C and D). This indicates that the key enzyme for histone lactylation, LDHA, is highly expressed in BCa in the TCGA database and suggests a poor prognosis. Additionally, we collected 15 pairs of cancer and adjacent tissues from BCa patients in our unit, extracted their proteins, and conducted Western blot experiments. The results suggested that RBM15, LDHA, H3K18la, and IGFBP3 were all highly expressed in BCa (Fig. 8E). Among them, 10 pairs of BCa tissue specimens underwent IHC experiments, and the staining positive areas were scored using the H-score. The results also indicated that H3K18la, RBM15, and IGFBP3 were all highly expressed in BCa (Fig. 8F). Furthermore, we compared the differences in the expression of H3K18la, RBM15, and IGFBP3 between 5 cisplatin-sensitive and 7 cisplatin-resistant BCa patients. The IHC assays showed that expression levels of H3K18la, RBM15, and IGFBP3 were higher in cisplatin-resistant patients (Fig. 8G). These results suggest that H3K18la, RBM15, and IGFBP3 are highly expressed in BCa clinical samples, and their expression levels were further elevated upon developing cisplatin resistance. A schematic diagram created with BioGDP.com illustrates the research mechanism of this article (Fig. 8H) [30].

## Discussion

In this study, we uncover a novel hypoxia–lactylation–RBM15–IGFBP3 axis that drives cisplatin resistance in BCa, providing mechanistic insights into the metabolic and epigenetic reprogramming underlying chemoresistance. Our findings demonstrate that hypoxia-induced lactate accumulation, mediated by LDHA, promotes H3K18la modification via the lactyltransferase KAT2B, leading to transcriptional activation of RBM15. RBM15 promotes the stability of IGFBP3 mRNA through m^6^A modification, thereby enhancing its expression. This cascade enhances DNA repair through activation of EGFR and DNA-PKcs, ultimately conferring resistance to cisplatin. These results not only elucidate a previously unrecognized link between hypoxia, lactylation, and m^6^A modification but also propose actionable therapeutic strategies to overcome cisplatin resistance in BCa.

Hypoxia is a well-established contributor to chemoresistance in solid tumors [31]. Previous studies in non-small cell lung cancer (NSCLC) and ovarian cancer have shown that hypoxia promotes downstream gene transcription through the HIF-1α pathway, which contributes to cisplatin resistance [32–35]. In BCa, hypoxia can also exert a role in promoting tumor progression and cisplatin resistance through the HIF-1α pathway [36,37]. In the KEGG enrichment analysis, we identified the HIF-1α signaling pathway, which plays a critical role in cellular response to hypoxia. Furthermore, the HIF-1α pathway may interact with the H3K18la/RBM15/IGFBP3 axis investigated in the present study, potentially collectively regulating cisplatin resistance in BCa. Specifically, in colorectal cancer, hypoxia-induced expression of H3K18la contributes to resistance against bevacizumab [12]. This study demonstrates that hypoxia also enhances cisplatin resistance in BCa through H3K18la-mediated mechanisms. Prior research has established that in BCa, H3K18la promotes tumor proliferation and migration by up-regulating LCN2 expression, and facilitates cisplatin resistance by enhancing the expression of YBX1 and YY1 [11,14]. Our findings further elucidate the critical role of hypoxia as a driver of histone lactylation, highlighting its significance in mediating cisplatin resistance in BCa and revealing a novel pathway for this phenomenon.

Current studies have identified several proteins as lactylation writers, including P300, CBP, AARS1, AARS2, KAT2A, KAT2B, KAT5, KAT8, and ATAT1 [8,12,13,16,28,38–44]. Many of these proteins also possess the ability to catalyze protein acetylation. While KAT6B was hypoxia-induced, overexpressing KAT6B did not affect H3K18la or RBM15, suggesting that it may lactylate non-H3K18 sites or other histones. Future studies will explore KAT6B’s targets. Previous research has established that KAT2B primarily catalyzes histone acetylation at H3K27ac and acetylation of certain nonhistone proteins [45,46]. One study reported that when the KAT2B protein has mutations at the Y616 and F617 positions to A616 and A617, its acetylation catalytic activity is lost [47]. Our study found that after mutating the amino acids at these 2 positions, its lactylation catalytic activity is also lost. Recent findings indicate that KAT2B can function as a lactylation writer, promoting the lactylation of the nonhistone protein EGR1 [41]. However, no studies have yet reported KAT2B’s role as a histone lactylation writer. Moreover, previous studies have found that knockdown of KAT2B can moderately inhibit the proliferation ability of BCa cells [48]. However, the role of KAT2B in cisplatin resistance of BCa has not been reported. This study is the first to demonstrate that under hypoxic conditions, KAT2B can promote the formation of histone H3K18la, thereby enhancing the expression of IGFBP3 and cisplatin resistance of BCa. Through a series of experiments investigating the interaction between KAT2B and Nala, we confirmed that the effects of Nala on BCa functions are mediated specifically by H3K18la rather than general lactate effects.

Multiple studies have found that there is a close connection between histone lactylation modification and mRNA or DNA modification. Although interactions between m^5^C modification, DNA methylation, and histone lactylation have been documented, our sequencing data revealed that H3K18la primarily interacts with m^6^A modification in BCa [49,50]. For instance, H3K18la promotes the progression of melanoma by facilitating the expression of the m^6^A reading protein YTHDF2 [51]. H3K18la aggravates diabetic retinopathy microvascular abnormalities by promoting the expression of the m^6^A eraser FTO [52]. IGF2BP2 increases the level of H3K18la by promoting the expression of the sugar metabolism-related gene ALDOA, thereby promoting liver fibrosis [53]. H3K18la promotes the expression of the m^6^A reading protein YTHDF1, facilitating the progression of pulmonary fibrosis [54]. H3K18la promotes the immune suppression effect of tumor-infiltrating myeloid cells by promoting the expression of METTL3 [55]. However, there is no research report on the regulation of H3K18la by m^6^A methyltransferase RBM15. RBM15 interacts with METTL3 and METTL14 to form the m^6^A writer complex and performs the function of mRNA methyltransferase [17]. RBM15 has 3 domains: RRM domain, SF-CC1 domain, and SPOC domain [21]. The SPOC domain of RBM15 can bind to other m^6^A writers and plays an important role in the formation of m^6^A modification. The SPOC domain is also crucial for maintaining the stability of downstream mRNA [56]. In this study, we also found that the SPOC domain plays a key role in the m^6^A modification level and expression of IGFBP3. Previous studies have found that RBM15 promotes the proliferation and migration of BCa [22,23]. However, no study has explored the role of RBM15 in cisplatin resistance of BCa. This study found that hypoxia promotes RBM15 expression through H3K18la, thereby promoting cisplatin resistance in BCa, revealing a new mechanism of cisplatin resistance in BCa.

IGFBP3, a p53-regulated gene involved in DNA damage repair, emerged as a central player in drug resistance [57]. In breast cancer, IGFBP3 promotes the phosphorylation and nuclear translocation of EGFR and DNA-PKcs by facilitating their binding. Phosphorylated DNA-PKcs induces NHEJ and confers resistance to etoposide [25]. In prostate cancer, IGFBP3 causes resistance to poly(adenosine diphosphate-ribose) polymerase (PARP) inhibitors through interaction with DNA-PKcs and EGFR [26]. In oral squamous cell carcinoma, IGFBP3 induces radiotherapy resistance through interaction with DNA-PKcs and EGFR [27]. In BCa, IGFBP3 has been reported to have a carcinogenic effect, but the relationship and mechanism between it and cisplatin resistance remain unclear [58,59]. Previous studies have also reported that the IGFBP3 protein contains a nuclear localization sequence (NLS), which facilitates its nuclear transport [60,61]. In our study, knockdown of IGFBP3 sensitized BCa cells to cisplatin, while its overexpression enhanced resistance, both in vitro and in vivo. These findings are consistent with prior studies implicating IGFBP3 in chemoresistance across various cancers. However, our work is the first to link IGFBP3 to hypoxia-induced histone lactylation, providing a mechanistic basis for its role in cisplatin resistance.

At present, for patients with advanced BCa who are resistant to cisplatin in clinical practice, chemotherapy combined with immunotherapy is the main treatment approach [62]. The targeted therapeutic drugs are mainly fibroblast growth factor receptor (FGFR) inhibitors Erdafitinib [63]. Although studies have found that the EGFR pathway plays an important role in the occurrence and development of BCa, EGFR inhibition combined with cisplatin can significantly inhibit tumor growth in nude mice, yet EGFRi has not shown effective effects on BCa patients who are resistant to cisplatin in clinical practice [64,65]. Therefore, the guideline does not consider EGFRi as the preferred treatment method for BCa patients who are resistant to cisplatin [66]. In previous studies, stiripentol combined with cisplatin has been found to be effective in treating cisplatin-resistant gastric cancer [28]. In BCa, LDHAi such as oxamate combined with cisplatin can also inhibit the growth of subcutaneous tumors of cisplatin-resistant BCa in nude mice [11]. In this study, it was found that LDHA and EGFR both play important roles in cisplatin resistance of BCa. LDHAi such as oxamate and stiripentol can both promote the cisplatin sensitivity of BCa, and stiripentol, as a drug for treating epilepsy in clinical practice, has better safety and application than oxamate [67]. Therefore, in this study, we combined stiripentol with gefitinib as EGFRi and gefitinib with stiripentol as LDHAi, which can promote the cisplatin sensitivity of cisplatin-resistant cells. Furthermore, while the absence of significant body weight changes among treatment groups suggests general tolerability, it is important to note that a more comprehensive safety assessment, including evaluation of biochemical parameters (e.g., liver and kidney function) and specific myocardial markers, was not conducted in this study. Future investigations will be necessary to fully characterize the safety profile of the administered compounds. If our treatment strategy is to be applied clinically, further large-scale clinical trials will be necessary for validating toxicity and pharmacokinetic interactions. Nevertheless, our findings indeed provide a new approach for treating cisplatin-resistant BCa. In addition, therapeutic approaches such as bacterial outer membrane vesicles (OMVs) and self-assembled nanomedicines offer novel strategies for the treatment of BCa [68,69].

While our study provides compelling evidence for the role of hypoxia-induced lactylation in cisplatin resistance, several questions remain. First, the precise mechanisms by which KAT2B catalyzes H3K18la and the potential involvement of other lactyltransferases warrant further investigation. Second, the broader impact of lactylation on the BCa epigenome and its interaction with other histone modifications remain to be explored. Finally, the translational potential of LDHA and EGFRi in clinical settings requires validation through prospective trials. Third, our axis likely coexists with other hypoxia-driven resistance mechanisms (e.g., reduced drug uptake and glutathione-mediated detoxification), underscoring hypoxia’s multifaceted role in chemoresistance. The complete mechanism by which hypoxia leads to cisplatin resistance in BCa still requires further investigation.

## Conclusion

In summary, our study elucidates a hypoxia–H3K18la–RBM15–IGFBP3 axis as a central mechanism of cisplatin resistance in BCa. By linking histone lactylation and m^6^A modification, this axis represents a multifaceted therapeutic target. Our findings not only advance the understanding of chemoresistance but also provide a rationale for combining metabolic and signaling inhibitors to improve outcomes in cisplatin-resistant BCa.

## Materials and Methods

### TCGA data collection and preprocessing

The gene expression profiles of BCa tissues were obtained from the TCGA database, comprising 401 BCa samples (TCGA-BLCA dataset). Raw data were normalized and standardized using R software. GSVA was performed with the GSVA package to evaluate the enrichment of tumor-related pathways. Tumor-associated gene sets were sourced from the MSigDB Hallmark database. GSVA quantified hallmark gene set activity for each sample, followed by univariate Cox proportional hazards regression to assess associations between gene sets and survival outcomes. Hazard ratios (HRs) and *P* values were calculated, with gene sets exhibiting *P* < 0.05 deemed statistically significant. A forest plot was generated to visualize high-risk pathways (HR > 1). WGCNA was conducted on transcript per million (TPM) values of protein-coding genes from the TCGA-BLCA dataset. The optimal soft-thresholding power (β = 9) was determined using the pickSoft Threshold function to ensure scale-free network topology. Modules containing fewer than 50 genes were excluded to enhance reliability. Module eigengenes (MEs) were correlated with GSVA scores via Pearson correlation analysis. LASSO Cox regression (λ = 0.064, selected by minimum cross-validation error) was applied to identify prognostic genes from hypoxia-associated modules. An HGS was constructed using regression coefficients, where the HGS score for each patient was calculated as follows: HGS score = Σ (coefficient × mRNA expression). Patients were stratified into high- and low-risk groups based on the median HGS score. Survival differences between groups were assessed using Kaplan–Meier analysis with log-rank tests (*P* < 0.05). Differentially expressed genes (DEGs) between risk groups were identified with the limma package (adjusted *P* < 0.05, |log_2_ fold change| > 1). Functional enrichment analysis of DEGs was performed using clusterProfiler for GO and KEGG pathways.

### Cell lines and cell culture

UM-UC-3 and T24 human BCa cells, along with the 293T human embryonic kidney cell line, were obtained from the Cell Bank of the Chinese Academy of Sciences (Shanghai, China). The cisplatin-sensitive T24 cell line was exposed to progressively increasing cisplatin concentrations to generate a cisplatin-resistant cell line (T24-R). T24 cells were exposed to stepwise cisplatin (1 to 30 μM over 6 months; pulsed 72-h treatment every 2 weeks). IC_50_ increased 13.3-fold versus parental T24. IC_50_ was rechecked every 10 passages; resistance remained stable. UM-UC-3 cells were cultured in minimum essential medium, T24 and T24-R cells in RPMI 1640 medium, and 293T cells in Dulbecco’s modified Eagle’s medium. All media were supplemented with 10% heat-inactivated fetal bovine serum (FBS) and maintained at 37 °C under 5% CO₂ in normoxic (20% O₂) or hypoxic (1% O₂) conditions.

### Human clinical samples

Fifteen paired fresh BCa tissues and adjacent nonmalignant bladder mucosal tissues, along with 5 cases of BCa tissue specimens that were sensitive to cisplatin and 7 cases that were resistant to cisplatin from patients undergoing radical cystectomy, were collected from the First Affiliated Hospital, Zhejiang University, School of Medicine (October 2021 to November 2024). Specimens were snap-frozen in liquid nitrogen or fixed in 37% formaldehyde. Informed consent and Ethics Committee approval were obtained prior to sample collection. Patient demographics are detailed in Table S1.

### Reagents and transfection

Overexpression plasmids (IGFBP3, KAT2B-WT, KAT2B-MUT, KAT6B, RBM15, RBM15 ΔSPOC, and vector) and short hairpin RNA (shRNA) plasmids (shNC, shIGFBP3-1, shIGFBP3-2, shRBM15-1, shRBM15-2, and shKAT2B) were sourced from MiaoLing Plasmid Platform. Transfections were performed using Polyplus transfection reagent for RNA duplexes and EZ Trans reagent (Life-iLab, China) for plasmids, following manufacturer protocols. The sequences of shRNA are listed in Table S2.

### Quantification of l-lactic acid

l-Lactic acid (LA) levels were measured using an LA Content Assay Kit (Solarbio) per the manufacturer’s instructions.

### Chromatin immunoprecipitation

A SimpleChIP Plus Enzymatic Chromatin IP Kit [Cell Signaling Technology (CST), #9004, USA] was used for ChIP-qPCR to assess H3K18la interactions with the RBM15 promoter, following the provided protocol. Primers used for qPCR are listed in Table S2.

### RNA isolation and RT-qPCR

Total RNA from BCa cells was extracted using the NcmSpin Cell/Tissue Total RNA Kit (NCMbio). cDNA synthesis was performed with StarScript III Reverse Transcriptase (GenStar), and qPCR was conducted on a Bio-Rad real-time PCR system using StarScript III SYBR Kit (GenStar). β-Actin served as the normalization control. Relative expression was calculated via the 2^−ΔΔCt^ method. Primer sequences are listed in Table S2.

### Cell viability assay and IC_50_ value determination

UM-UC-3 or T24 cells (3,000 per well) were seeded in 96-well plates and treated with cisplatin, Nala, LDHAi (like oxamate and stiripentol), or EGFRi (like gefitinib) at varying concentrations. Cell viability was assessed every 24 h using CCK-8 (NCMbio). IC_50_ values were measured after treatment with cisplatin for 48 h. Cell viability and IC_50_ values were calculated using GraphPad Prism 8.

### Colony formation assay

Cells (500 per well) were seeded in 6-well plates and treated as indicated. Colonies were fixed with methanol, stained with 0.1% crystal violet, and counted after 10 to 14 d.

### Analysis of apoptosis

Apoptotic rates were determined using an Annexin V-FITC/PI Apoptosis Detection Kit (BD Biosciences). Cells were analyzed via BD FACSCalibur and FlowJo software.

### Protein IP

IP was performed using a Co-IP Kit (Beyotime). Lysates from 1 × 10^7^ cells were incubated overnight at 4 °C with antibody-conjugated protein A/G magnetic beads. Beads were boiled in loading buffer for subsequent analysis.

### MeRIP-quantitative real-time PCR

MeRIP-qPCR was performed using the Magna MeRIP m^6^A Kit (Merck Millipore) according to manufacturer guidelines. Briefly, total RNA isolated from UM-UC-3 cell lines was fragmented to ~100 nucleotides. Fragmented RNA underwent IP for 2 h at 4 °C with magnetic beads conjugated to either m^6^A antibody or IgG. Bound RNA was eluted and purified using a Qiagen RNA purification kit. Purified RNA was analyzed by real-time qPCR (RT-qPCR) with primers listed in Table S2. M^6^A enrichment was normalized to input.

### RIP-quantitative real-time PCR

RIP-qPCR was performed using the PureBinding RNA Immunoprecipitation Kit (Geneseed). Lysates of UM-UC-3 cells were centrifuged, and supernatants were incubated overnight at 4 °C with magnetic beads conjugated to 5 μg of either RBM15 antibody or IgG. Bead-bound complexes were then treated with proteinase K, followed by RNA isolation and purification for subsequent RT-qPCR analysis.

### Dual-luciferase reporter assay

Plasmids containing either wild-type (IGFBP3-WT) or mutant (IGFBP3-MUT) target regions were synthesized by MiaoLing Plasmid Platform. UM-UC-3 seeded in 96-well plates were transfected with the respective reporter plasmid. Relative luciferase activity was measured using the Dual Luciferase Reporter Assay Kit (Vazyme).

### Western blot assay

Cells or tissues were first lysed to extract the total proteins. The protein concentration was quantified, and the samples were then mixed with loading buffer. Subsequently, the samples were heat-denatured to ensure complete denaturation of the proteins. The denatured samples were loaded onto a polyacrylamide gel, and an electric current was applied to separate the proteins according to their molecular weight. Following electrophoresis, the separated proteins were transferred from the gel onto a polyvinylidene difluoride (PVDF) membrane via electroblotting, which immobilizes them for subsequent steps. The membrane was then blocked by incubation with nonfat milk to prevent nonspecific antibody binding. After blocking, the membrane was incubated with a specific primary antibody. Following primary antibody incubation, the membrane was washed to remove unbound antibodies and then incubated with an enzyme-conjugated secondary antibody. After another washing step, a chemiluminescent substrate was added, which produces light upon reaction with the secondary antibody. Finally, the emitted light signal was captured using a digital imager to visualize the target protein bands. All antibodies used in this procedure are listed in Table S3.

### Dot blot assay

Total RNA was extracted from BCa cells or tissues and adjusted to a concentration of 50 ng/μl using ribonuclease-free water. Following denaturation to eliminate secondary structures, the RNA samples were treated with ice-cold 20× saline sodium citrate (SSC; Sigma-Aldrich). The samples were then loaded onto an N+ membrane (GE Healthcare) using a dot blot apparatus (Bio-Rad). After loading, the membrane was ultraviolet-cross-linked and stained with methylene blue (Sigma-Aldrich). The membrane was subsequently washed to remove the methylene blue stain. It was then incubated in a blocking solution, followed by incubation with an anti-m^6^A primary antibody. After washing to remove unbound primary antibody, the membrane was incubated with a secondary antibody. Another washing step was performed, after which a chemiluminescent substrate was added to visualize the bound secondary antibody. Positive signals appeared as detectable dots on the membrane.

### RNA sequencing

Total RNA was extracted with TRIzol. Libraries were prepared and sequenced by LC-Bio. DEGs were analyzed using OmicStudio tools (https://www.omicstudio.cn/tool) for heatmap, volcano plot, and KEGG pathway enrichment. An interactive tool was used for comparing lists with Venn’s diagrams (https://bioinfogp.cnb.csic.es/tools/venny/index.html).

### Animal experiments

BALB/c nude mice (4-week-old males) were subcutaneously injected with 2 × 10^6^ BCa cells. After 1 week, mice received intraperitoneal cisplatin (3 mg/kg), stiripentol (150 mg/kg), gefitinib (10 mg/kg), or phosphate-buffered saline (PBS) twice weekly. Tumor volume (*V* = 0.52 × width^2^ × length) was monitored weekly. Mice were euthanized following institutional guidelines of the First Affiliated Hospital, School of Medicine, Zhejiang University.

### Immunofluorescence

Cells (4 × 10^4^ per well) were fixed with 4% paraformaldehyde, blocked with 5% FBS, and incubated overnight with anti-IGFBP3 (1:100, Proteintech), anti-p-DNA-PKcs (1:100, Diagbio), or anti-p-EGFR (1:100, Diagbio). Alexa Fluor-conjugated secondary antibodies (1:1,000, CST) and 4′,6-diamidino-2-phenylindole (DAPI) (Beyotime) were used for visualization via confocal microscopy.

### Immunohistochemistry

Tissues were fixed in 4% paraformaldehyde at 4 °C for 16 to 18 h and dehydrated. Paraffin-embedded sections (4 μm) were incubated with anti-RBM15 (1:200, CST), anti-IGFBP3 (1:500, Proteintech), or anti-H3K18la (1:500, PTMbio) antibodies overnight. Secondary antibodies (1:2,000, CST) and 3,3′-diaminobenzidine (DAB) staining were applied for analysis. The percentage of positively stained cells (frequency) was scored on a scale of 0 (no staining) to 100 (all staining). Staining intensity was graded as 0 (no staining) to 3 (strongly positive). The IHC score, ranging from 0 to 300, was calculated by multiplying the frequency score by the intensity score.

### Statistical analysis

Data are presented as mean ± SEM. Group differences were analyzed using Student’s *t* test or chi-square test (SPSS 25.0). A 2-tailed *P* < 0.05 was considered statistically significant.

## Data Availability

The datasets used and/or analyzed in this article were included within the article and the additional files. Please contact the corresponding author for data requests.
