# Supplementary material for: Hypoxia-Induced Histone Lactylation Drives Cisplatin Resistance in Bladder Cancer by Promoting RBM15-Dependent m6A Methylation of IGFBP3
Source: Research (Wash D C). 2025 Nov 5;8:0970. doi: 10.34133/research.0970 (PMC12586852; doi:10.34133/research.0970)
Supplement: Supplementary 1 — Figs. S1 to S7 Tables S1 to S3 [file research.0970.f1.zip › research.0970f1.docx]

**Supplementary Tables**

**Table S1. Patient information of clinical samples.**

| **No.** | **Age** | **Gender** | **Pathologic diagnosis** | **TNM Stage** | **Resistent** |
| --- | --- | --- | --- | --- | --- |
| 1 | 75 | Male | Urothelium carcinoma | pT2bN0Mx | NO |
| 2 | 68 | Male | Urothelium carcinoma | pT2N0Mx | NO |
| 3 | 70 | Male | Urothelium carcinoma | pT1N0Mx | NO |
| 4 | 61 | Male | Urothelium carcinoma | pT3aN2Mx | NO |
| 5 | 71 | Male | Urothelium carcinoma | pT1N0Mx | NO |
| 6 | 57 | Male | Urothelium carcinoma | pTaN0Mx | NO |
| 7 | 77 | Male | Urothelium carcinoma | pT4N0Mx | NO |
| 8 | 71 | Male | Urothelium carcinoma | pT1N0Mx | NO |
| 9 | 76 | Male | Urothelium carcinoma | pT2aN0Mx | NO |
| 10 | 74 | Male | Urothelium carcinoma | pT3aN1Mx | NO |
| 11 | 72 | Male | Urothelium carcinoma | pT1N0Mx | NO |
| 12 | 64 | Male | Urothelium carcinoma | pT2bN0Mx | NO |
| 13 | 77 | Female | Urothelium carcinoma | pT3aNxMx | NO |
| 14 | 59 | Male | Urothelium carcinoma | pT1N0Mx | NO |
| 15 | 72 | Male | Urothelium carcinoma | pT1N0Mx | NO |
| 16 | 78 | Male | Urothelium carcinoma | pT4N1Mx | YES |
| 17 | 77 | Male | Urothelium carcinoma | pT4N+Mx | YES |
| 18 | 76 | Male | Urothelium carcinoma | pT4N0Mx | YES |
| 19 | 65 | Male | Urothelium carcinoma | pT4aNxMx | YES |
| 20 | 72 | Male | Urothelium carcinoma | pT4aNxMx | YES |
| 21 | 69 | Male | Urothelium carcinoma | pT4aNxMx | YES |
| 22 | 78 | Male | Urothelium carcinoma | pT3aNxMx | YES |
| 23 | 54 | Male | Urothelium carcinoma | pT1N0Mx | NO |
| 24 | 59 | Male | Urothelium carcinoma | pT1N0Mx | NO |
| 25 | 77 | Female | Urothelium carcinoma | pT3aNxMx | NO |
| 26 | 82 | Male | Urothelium carcinoma | pT3aN0Mx | NO |
| 27 | 69 | Female | Urothelium carcinoma | pT4aN0Mx | NO |

**Table S2. The oligonucleotides used in this study.**

| **Primers for qRT-PCR** | | |
| --- | --- | --- |
| **Target** | **Forward primer (5’-3’)** | **Reverse primer (5’-3’)** |
| IGFBP3 | AGAGCACAGATACCCAGAACT | GGTGATTCAGTGTGTCTTCCATT |
| β-actin | CTCCATCCTGGCCTCGCTGT | GCTGTCACCTTCACCGTTCC |
| RBM15 | ACGACCCGCAACAATGAAG | GGAAGTCGAGTCCTCACCAC |
| IGFBP3-m^6^A | CTGCCGCAAGGTTAATGTGG | AATATAAATCGAGGCTGTAGCCAG |
| RBM15-ChIP | AAGGCTGGGAGGAATGCTTA | TGCTGCCTGTCTTCCTTCTC |

| **Sequence of shRNA** | | |
| --- | --- | --- |
| **Name** | **Sense (5’-3’)** | **Anti-sense (5’-3’)** |
| shNC | GTTCTCCGAACGTGTCACGT | ACGTGACACGTTCGGAGAAC |
| shIGFBP3-1 | CAAAGATAATCATCATCAAGA | TCTTGATGATGATTATCTTTG |
| shIGFBP3-2 | GCTGGTGTGTGGATAAGTATG | CATACTTATCCACACACCAGC |
| shRBM15-1 | CCTCAACAATGAAAGGAAATT | TTTCCTTTCATTGTTGAGGTT |
| shRBM15-2 | GAAACAGGATGGAGGGACATT | TGTCCCTCCATCCTGTTTCTT |
| shKAT2B | GCAGACTTACAGCGAGTCTTT | GCAGATACCAAACAAGTTTAT |

**Table S3. Antibodies used in this study.**

| **Reagent or Resouce**  **REAGENT or RESOURCE** | **Source** | **Identifier** |
| --- | --- | --- |
| Anti-L-Lactyl Lysine Rabbit mAb | PTMBio | PTM-1401RM |
| Anti-L-Lactyl-Histone H3 (Lys18) Rabbit mAb | PTMBio | PTM-1406RM |
| Anti-L-Lactyl-Histone H3 (Lys18) Rabbit mAb-ChIP Grade | PTMBio | PTM-1427RM |
| Anti-PCAF(KAT2B) Rabbit mAb | PTMBio | PTM-5711 |
| Phospho-Histone H2A.X (Ser139) Recombinant antibody | Proteintech | **No.** 83307-2-RR |
| Beta Actin Monoclonal antibody | Proteintech | **No.** 66009-1-Ig |
| Histone H3 Polyclonal antibody | Proteintech | **No.** 17168-1-AP |
| IGFBP3 Monoclonal antibody | Proteintech | **No.** 68402-1-Ig |
| DYKDDDDK tag Monoclonal antibody | Proteintech | **No.** 66008-4-Ig |
| LDHA-Specific Polyclonal antibody | Proteintech | **No.** 19987-1-AP |
| DNA PKcs Recombinant Rabbit mAb | Diagbio | db15780 |
| Phospho-DNA PKcs (Ser2056) Recombinant Rabbit mAb | Diagbio | db13966 |
| EGFR Recombinant Rabbit mAb | Diagbio | db13403 |
| Phospho-EGFR (Tyr1068) Recombinant Rabbit mAb | Diagbio | db14353 |
| IGFBP3 Recombinant Rabbit mAb  53BP1 (DGR14147) Rabbit mAb | Diagbio  Diagbio | db14522  db15410 |
| RBM15 (E8Y8A) Rabbit mAb | CST | 60386 |
| N6-Methyladenosine (m6A) (D9D9W) Rabbit mAb  Anti-Phospho-Histone H2A.X (Ser139) Mouse mAb | CST  epizyme | 56593  98M19K61 |

**Supplementary Figures**

**
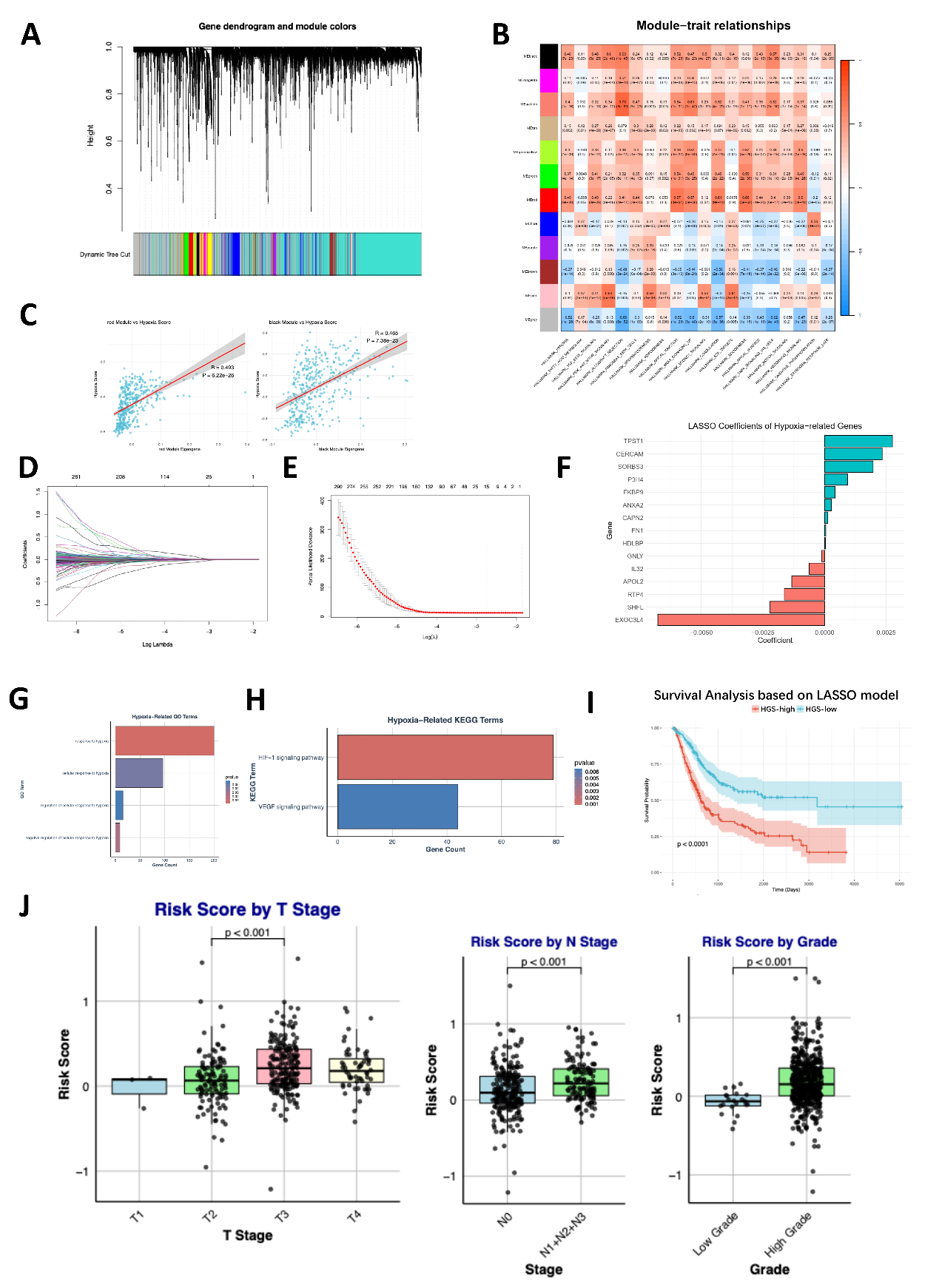
**

**Figure S1. Hypoxia-related gene analysis `in bladder cancer using TCGA data.**
(A) Co-expression module clustering dendrogram of 401 BCa samples generated by WGCNA. (B) Heatmap depicting correlations between module eigengenes (MEs) and cancer hallmarks. (C) Significant association of the red and black co-expression modules with hypoxia scores (Pearson’s r > 0.6, p < 0.001). (D) LASSO coefficient profiles of hypoxia-related differentially expressed genes. (E) Ten-fold cross-validation for optimal penalty parameter (λ) selection in LASSO regression (λ.min = 0.064). (F) LASSO regression coefficients of the 16-gene HGS. (G, H) Functional enrichment analysis of DEGs between HGS-high and HGS-low groups: (G) GO terms. (H) KEGG pathways. (I) Kaplan-Meier OS curves for HGS-high versus HGS-low groups (log-rank p < 0.001). (J) Association between HGS scores and advanced clinicopathological features (T/N stage, pathological grade).


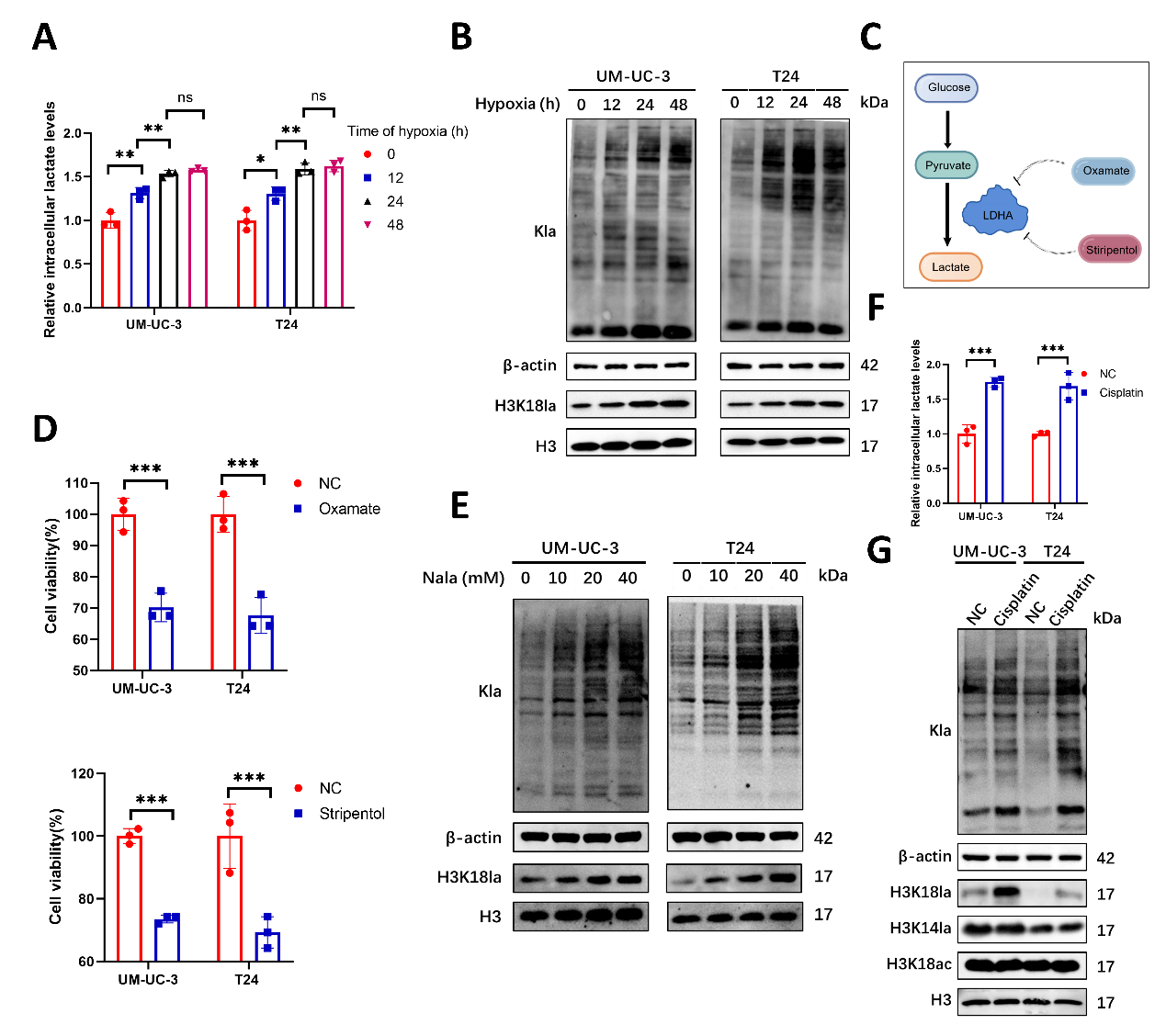


**Figure S2. L-lactate and lactylation levels in bladder cancer cells.**
(A, B) L-lactate levels (A) and lactylation levels (B) in bladder cancer cells following hypoxia exposure for 0, 12, 24, and 48 hours. (C) Schematic of glycolysis highlighting LDHA inhibition by oxamate and stiripentol. (D) Cell viability of bladder cancer following treatment with LDHA inhibitors oxamate (20 mM) and stiripentol (100 μM). (E) Lactylation levels in bladder cancer cells following 0, 10, 20, and 40 mM Nala treatment for 24h. (F) L-lactate levels in bladder cancer cells after cisplatin treatment (2μM). (G) Western blot analysis of lactylation levels in cisplatin-treated cells (2μM). Data: mean ± SD (n = 3). **p* < 0.05; ***p* < 0.01; ****p* < 0.001; ns, not significant.


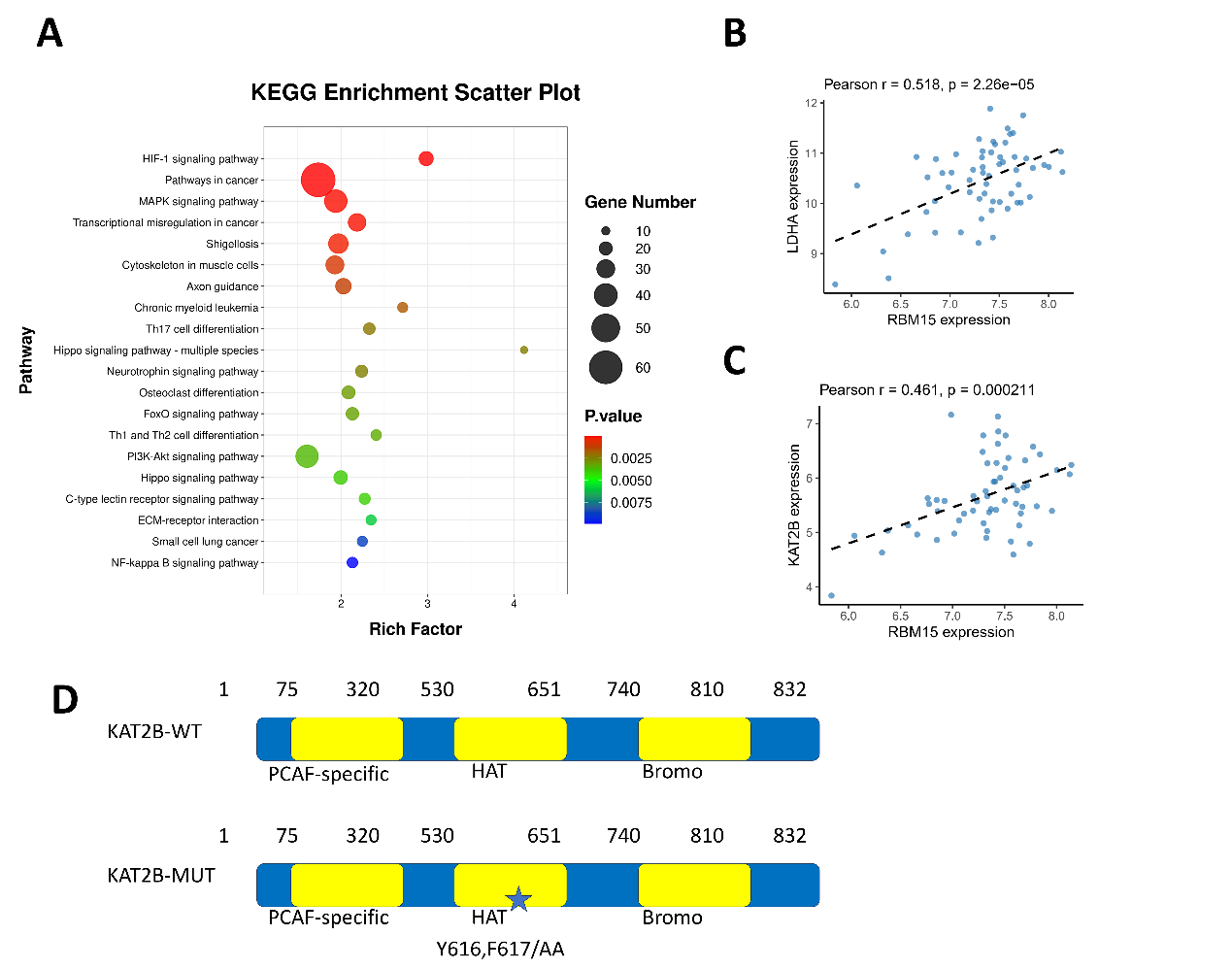


**Figure S3. KEGG pathways and TCGA database analysis**
(A) KEGG pathways enriched among hypoxia-upregulated genes and H3K18la downstream genes (B) TCGA database showing the expression relationship between LDHA and RBM15 in bladder cancer patient samples. (C) The TCGA database showing the expression relationship between KAT2B and RBM15 in bladder cancer patient samples. (D)Schematic diagram of KAT2B-WT and KAT2B-MUT protein.


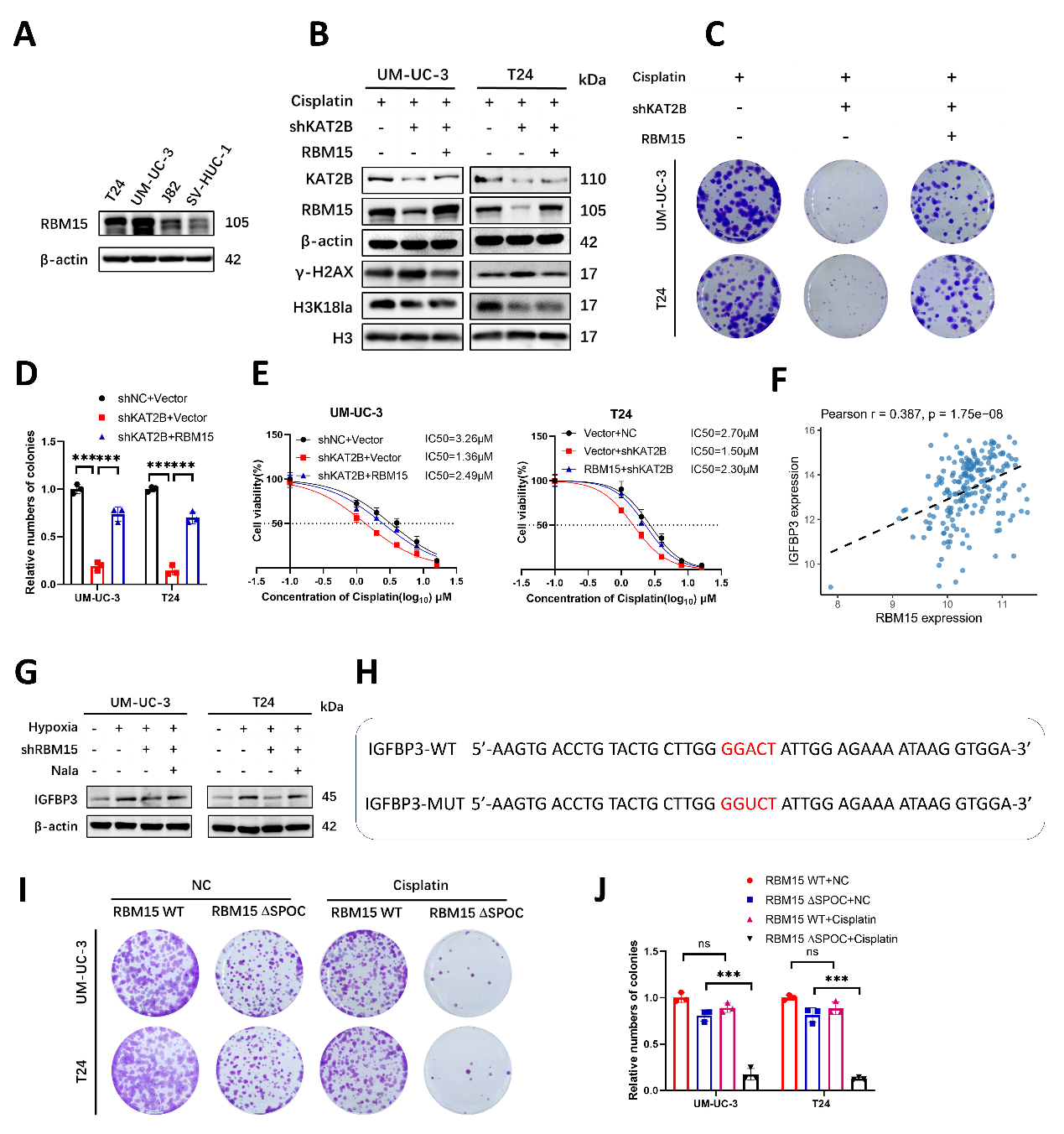


**Figure S4. RBM15-related rescue experiments**

(A) Western blot analysis of RBM15 in T24, UM-UC-3, J82 and SV-HUC-1 cells. (B) Western blot analysis of γ-H2AX, RBM15 and KAT2B in cisplatin-treated cells (1μM). (C, D) Colon formation assays of RBM15/KAT2B interplay of cisplatin-treated cells (1μM) (C). Quantification of numbers of colony (D). (E) IC50 values of RBM15/KAT2B interplay of cisplatin-treated cells. (F) Positive correlation between IGFBP3 and KAT2B mRNA of BCa in TCGA database. (G) Western blot analysis of IGFBP3 in hypoxia-treated cells (1% O₂). (H) Schematic diagram of IGFBP3 -WT and IGFBP3-MUT Sequence. (I, J) Colon formation assays of RBM15 WT and RBM15 ΔSPOC overexpressed cells with cisplatin-treated (1μM) (I). Quantification of numbers of colony (J). ****p* < 0.001; ns, not significant.


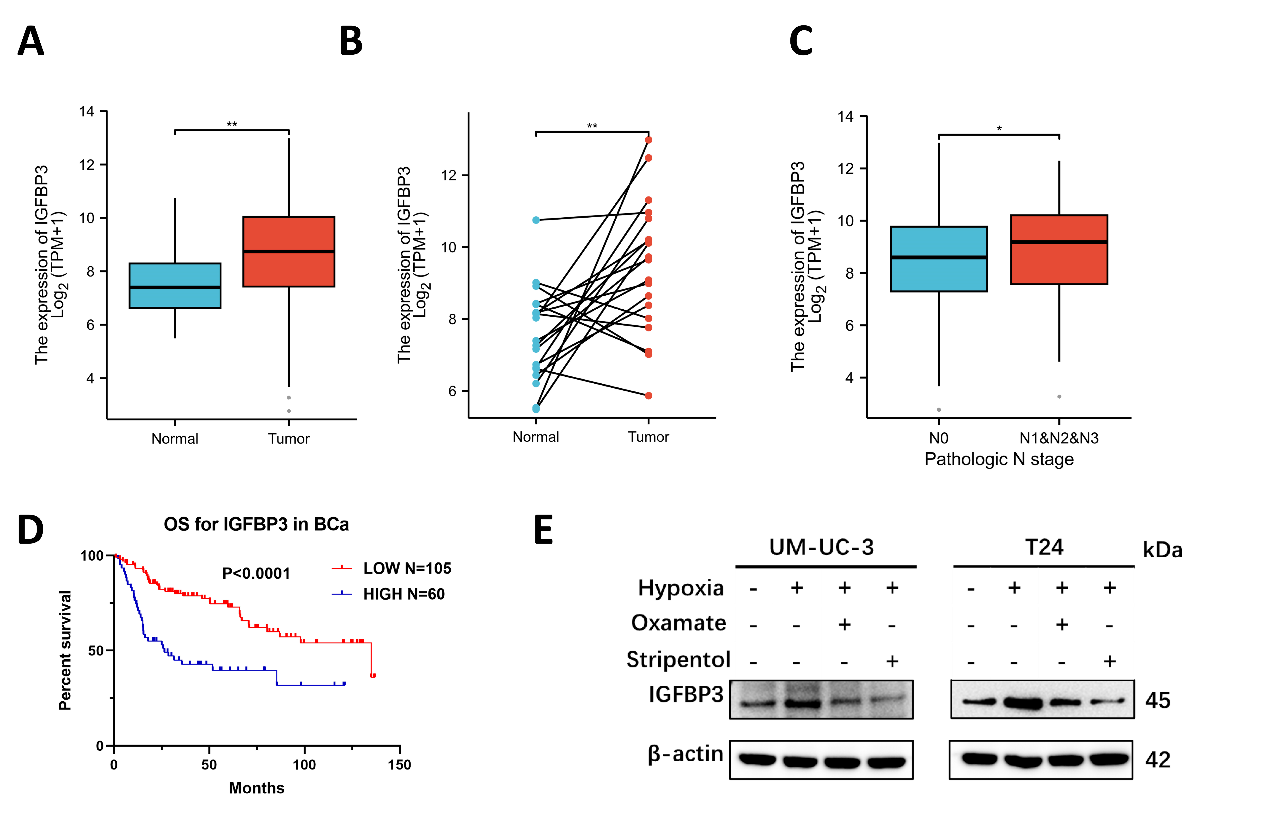


**Figure S5. The expression pattern of IGFBP3 in bladder cancer**

(A) IGFBP3 mRNA expression in normal vs. tumor bladder tissues in TCGA database. (B) IGFBP3 mRNA in paired tumor/normal samples in TCGA database. (C) IGFBP3 mRNA levels in patients with/without lymph node metastasis in TCGA database. (D) Kaplan-Meier analysis linking high IGFBP3 expression to reduced overall survival in bladder cancer patients. (E) Western blot of IGFBP3 in hypoxia-treated cells (1% O₂). **p* < 0.05; ***p* < 0.01; ns, not significant.


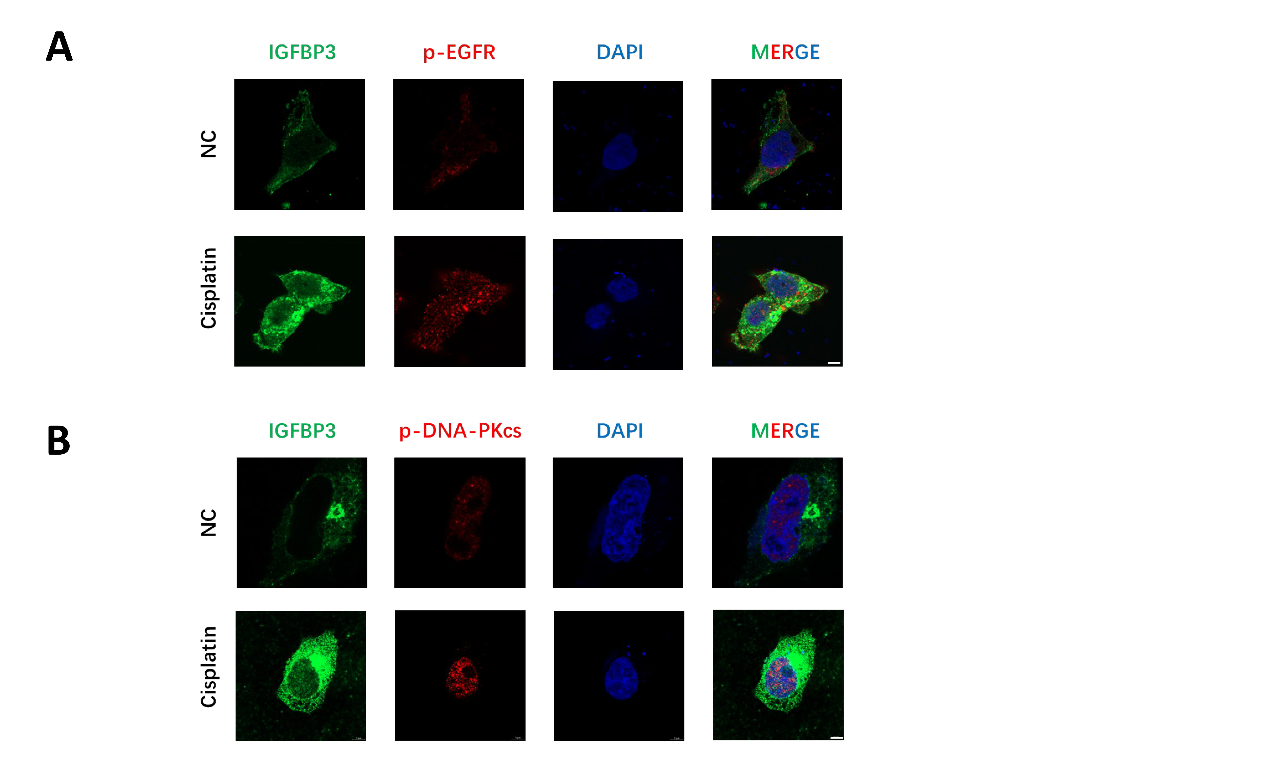


**Figure S6. Cisplatin induces nuclear colocalization of IGFBP3 with DNA repair markers.**
(A) Immunofluorescence of IGFBP3 (green), p-EGFR (Tyr1068, red), and DAPI (nuclei, blue) in UM-UC-3 cells with/without cisplatin treatment. (B) Immunofluorescence of IGFBP3 (green), p-DNA-PKcs (Thr2609, red), and DAPI (blue) in cisplatin-treated cells. Scale bars: 5 μm.


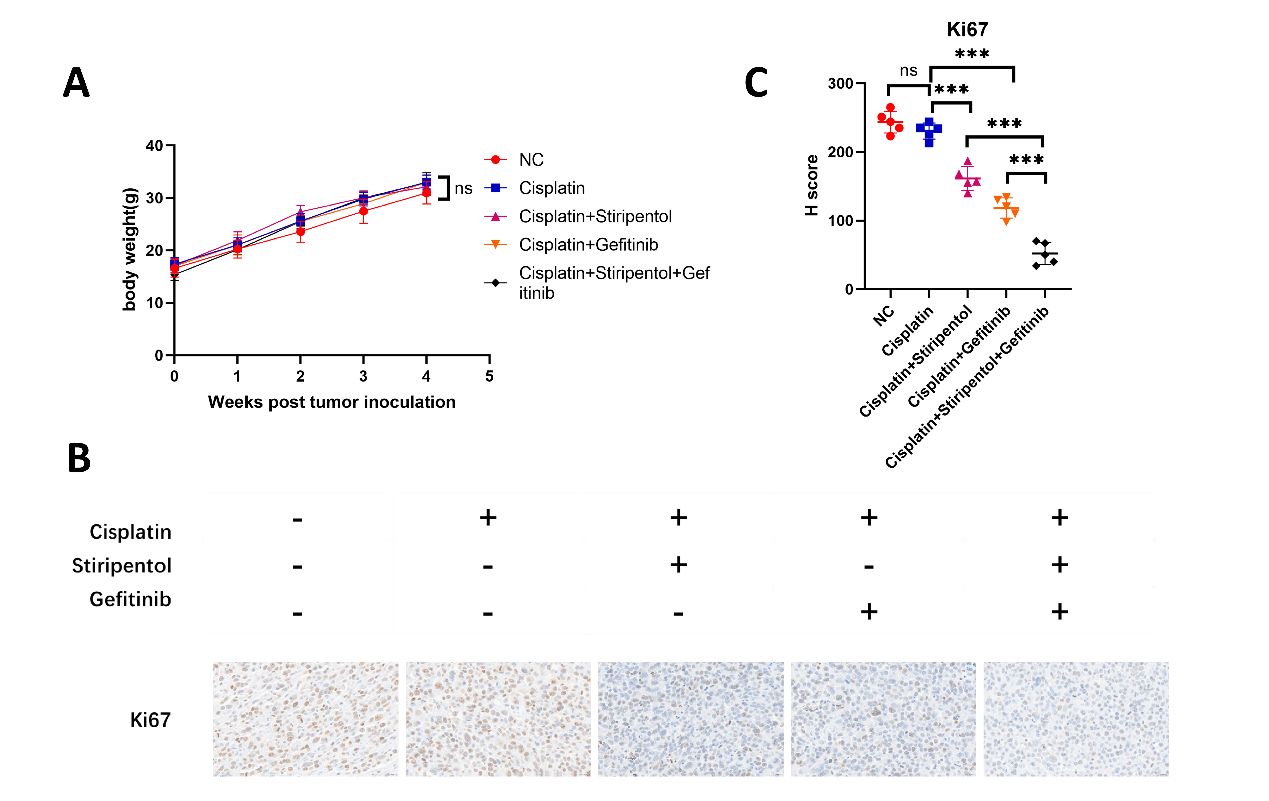


**Figure S7. Subcutaneous tumors in nude mice.**

(A) Body weight of mice. (B) IHC staining of Ki67 in subcutaneous tumors. (C) H score of Ki67 in subcutaneous tumors. ****p* < 0.001; ns, not sig
